# Visible-light-mediated sulfonylation of anilines with sulfonyl fluorides

**DOI:** 10.3389/fchem.2023.1267223

**Published:** 2023-08-25

**Authors:** Xin-Qing Li, Qian-Qian Liao, Jun Lai, Yuan-Yue Liao

**Affiliations:** ^1^ Department of Pharmacy, Ganzhou People’s Hospital, The Affiliated Ganzhou Hospital of Nanchang University, Ganzhou, China; ^2^ Department of Pharmacy, People’s Hospital of Guilin, Guilin, China

**Keywords:** sulfonylation, photoredox, radical, sulfonyl fluoride, aniline

## Abstract

Sulfonylaniline motif plays an important role in pharmaceutical sciences. Developed methods towards this structure are typically lack of good modifiability and stability. In this study, visible-light-mediated sulfonylation of aniline using sulfonyl fluoride as a modifiable and stable sulfonylation reagent is described. A variety of substituted sulfonylanilines were synthesized under mild reaction conditions with moderate to good efficiency. The example of late-stage sulfonylation highlighted the advantage of using sulfonyl fluoride as a sulfonylation reagent. In addition, the crucial influence of counterions on the photocatalyst observed in this system would inspire further research on the photochemistry of sulfonyl fluoride.

## 1 Introduction

**FIGURE 1:**
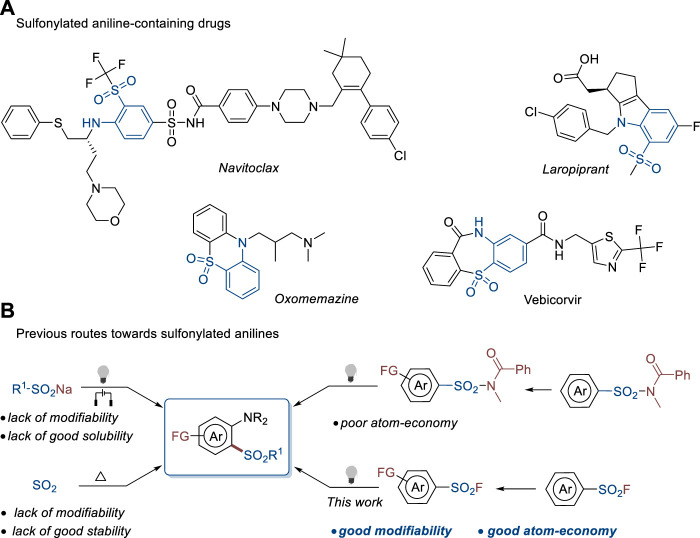
Sulfonylaniline-containing drugs and their construction methods. **(A)** Sulfonylated aniline-containing drugs. **(B)** Previous routes towards sulfonylated anilines.

Both sulfonyl and amine groups are typically considered privileged skeletons in medicinal chemistry for the discovery of biologically active compounds since more than 60% of bioactive molecules discovered in the past 40 years include amine units, while the percentage of the sulfonyl group is 3.1 (Ertl et al., 2020). The sulfonylaniline motif which combines these two groups also widely exists in diverse drugs, such as the Bcl-2 protein inhibitor *navitoclax* (Souers et al., 2013), DP receptor antagonist *laropiprant* (Sturino et al., 2007), histamine H1-receptor blocker *oxomemazine* (Lee et al., 1994), and hepatitis B virus core protein inhibitor *vebicorvir* (Sulkowski et al., 2022) (Figure 1A). Therefore, the development of versatile, efficient, and atom economic routes toward diverse sulfonylated anilines is highly important for both organic synthesis and pharmaceutic sciences.

Recently, several research groups have achieved outstanding results on the sulfonylation of anilines (Figure 1B). Johnson et al. (2018) reported the reaction of anilines and sulfinate salts mediated by visible light in the presence of an external oxidant. Wu et al. (2019), Lu et al. (2019), Nikl et al. (2019) reported the electrochemical synthesis of 2-sulfonylanilines with sulfinate salts and anilines. Sulfur dioxide has been widely used as a sulfonylation reagent in recent years (Emmett et al., 2015; Qiu et al., 2018; Ye et al., 2019; Zhang et al., 2023). In addition, a three-component reaction of anilines, DABCO(SO_2_)_2_, and aryldiazonium tetrafluoroborates was developed by the Wu group to construct sulfonylated anilines in high efficiency under metal-free conditions (Zhou et al., 2018). Several research groups demonstrated that sulfonamides could be used as sulfonylation reagents (Luo et al., 2021; Du et al., 2022; Zhen et al., 2022) to perform sulfonylation of anilines (Xu et al., 2022) and generated diverse complex desired products. Despite these achievements, there are obvious shortages in these methodologies. Using sulfinate salts as substrates usually requires an external oxidant. In addition, sulfinate salts have poor solubility in organic solvents, which hinders the modification of the substrates to construct complex products. The aryldiazonium salts used in the capture of SO_2_ have poor stability and also lack modifiability. Although sulfonamides are stable and can be modified to synthesize complex products, the released amide group underlines the poor atom economy of this method. Therefore, the exploration of novel strategies and sulfonylation reagents to construct sulfonylated anilines is of high demand in both synthetic chemistry and drug discovery.

Sulfonyl fluoride, which is readily available in the form of aryl halide (Davies et al., 2017), sulfonyl chloride (Dong et al., 2014), sulfonic acid (Jang et al., 2010), sulfinate salt (Banks et al., 1996), and thiophenol (Wright et al., 2005), is usually used in chemical probes (Jones et al., 2018; Mortenson et al., 2018; Martin-Gago et al., 2019) and polymer materials (Gao et al., 2017; Durie et al., 2018). However, it has rarely been used as a sulfonylation reagent for functionalization of the C–H bond. The successful examples required harsh reaction conditions to proceed with Friedel–Crafts substitution of arenes in the presence of AlCl_3_ (Hyatt et al., 1984). Because of the inertness of the SO_2_–F bond (Chinthakindi et al., 2018), sulfonyl fluoride can survive under diverse functionalization reaction conditions, including Suzuki–Miyaura cross-coupling (Chinthakindi et al., 2016), Heck (Qin et al., 2016), Stille (Hett et al., 2015), and Sonogashira reactions (Fadeyi et al., 2016). Therefore, we explored whether sulfonyl fluoride could be used as a stable, modifiable, and atom economic sulfonylation reagent to react with aniline under mild reaction conditions. Herein, we report a simple and mild protocol to construct sulfonylated aniline by a visible-light-mediated reaction of sulfonyl fluoride and aniline via a radical process.

## 2 Results and discussion

4-Methylbenzenesulfonyl fluoride **1a** and *N*,*N*,4-trimethylaniline **2a** were used to optimize the reaction conditions. No or only trace amounts of the product were observed using methylene blue or 4-CzIPN as the photocatalyst in MeCN under the irradiation of blue light (Table 1, entries 1 and 2). Ru(ppy)_3_ provided the desired product **3a** in 10% yield (entry 3). Ir[(dFCF_3_ppy)_2_(dtbbpy)]PF_6_, Ir[(dFCF_3_ppy)_2_(bpy)]PF_6_, and Ir [(ppy)_2_(dtbbpy)]PF_6_ could not afford better results (entries 4–6). Ir[(ppy)_2_(dtbbpy)]PF_6_ showed slightly higher efficiency, furnished **3a** in a 21% yield. Interestingly, we found that the counterion of the photocatalyst had a crucial influence on the efficiency. Ir[(ppy)_2_(dtbbpy)]Cl, which was rarely used in the organic synthesis, dramatically increased the yield of **3a** to 46%. Other organic solvents, such as THF, DMSO, DCE, and CH_3_OH, exhibited a much inferior efficiency than MeCN. In an attempt to develop an environment-benign strategy, water with a combination of diverse surfactants was used as the solvent. However, only a 32% yield was obtained as the best result in the presence of Tween 40 (entries 13–17). Finally, various organic and inorganic bases were also screened. We found that the weak base KF provided a 68% yield of **3a** (entry 20). In addition, NaHCO_3_ gave the best result with an 82% yield (entry 23). Only low yields (0%–25%) were obtained using stronger bases, including K_3_PO_4_, CsF, DABCO, and Na_2_CO_3_, probably because of the hydrolysis of sulfonyl fluorides (Chinthakindi et al., 2016).

**TABLE 1:** Optimization of the reaction conditions^a^.
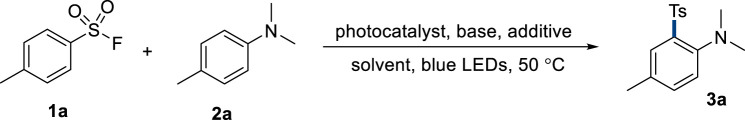
.

Entry	Photocatalyst	Solvent	Additive	Base	Yield (%)
1	Methylene blue	MeCN	-	-	nd
2	4-CzIPN	MeCN	-	-	Trace
3	Ru(ppy)_3_	MeCN	-	-	10
4	Ir[(dFCF_3_ppy)_2_(dtbbpy)]PF_6_	MeCN	-	-	10
5	Ir[(dFCF_3_ppy)_2_(bpy)]PF_6_	MeCN	-	-	Trace
6	Ir[(ppy)_2_(bpy)]PF_6_	MeCN	-	-	9
7	Ir[(ppy)_2_(dtbbpy)]PF_6_	MeCN	-	-	21
8	Ir[(ppy)_2_(dtbbpy)]Cl	MeCN	-	-	46
9	Ir[(ppy)_2_(dtbbpy)]Cl	THF	-	-	7
10	Ir[(ppy)_2_(dtbbpy)]Cl	DMSO	-	-	Trace
11	Ir[(ppy)_2_(dtbbpy)]Cl	DCE	-	-	20
12	Ir[(ppy)_2_(dtbbpy)]Cl	CH_3_OH	-	-	15
13	Ir[(ppy)_2_(dtbbpy)]Cl	H_2_O	-	-	6
14	Ir(ppy)_2_(dtbbpy)]Cl	H_2_O	Span 60	-	21
15	Ir[(ppy)_2_(dtbbpy)]Cl	H_2_O	Tween 40	-	32
16	Ir[(ppy)_2_(dtbbpy)]Cl	H_2_O	Tween 60	-	18
17	Ir [(ppy)_2_(dtbbpy)]Cl	H_2_O	Bu_4_NBF_4_	-	11
18	Ir[(ppy)_2_(dtbbpy)]Cl	MeCN	-	K_3_PO_4_	7
19	Ir[(ppy)_2_(dtbbpy)]Cl	MeCN	-	CsF	25
20	Ir[(ppy)_2_(dtbbpy)]Cl	MeCN	-	KF	68
21	Ir[(ppy)_2_(dtbbpy)]Cl	MeCN	-	Na_2_CO_3_	14
22	Ir[(ppy)_2_(dtbbpy)]Cl	MeCN	-	DABCO	nd
23	Ir[(ppy)_2_(dtbbpy)]Cl	MeCN	-	NaHCO_3_	82

^a^ Reaction conditions: **1a** (0.18 mmol, 1.8 equivalent), **2a** (0.1 mmol, 1.0 equiv), photocatalyst (5 mol%), additive (0.3 mmol, 3.0 equivalent), base (0.18 mmol, 1.8 equivalent), solvent (1.0 mL), 50°C, 30-W blue LEDs, and 12 h. GC yield with dodecane as the internal. nd = not detected.

**FIGURE 2:**
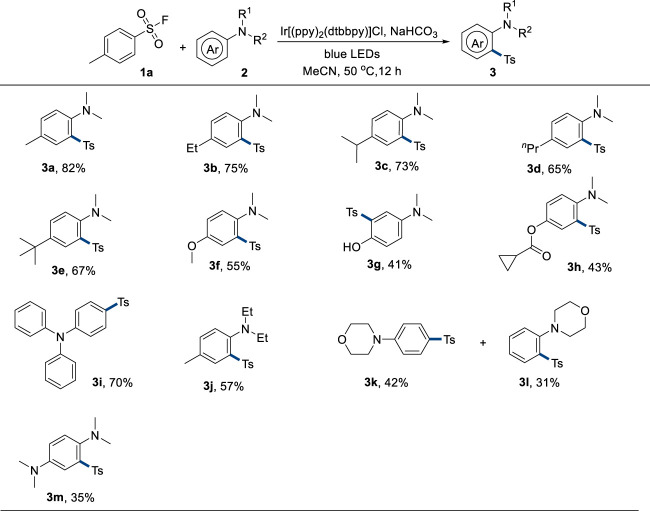
Scope of anilines. Reaction conditions: **1a** (0.36 mmol, 1.8 equivalent), **2** (0.2 mmol, 1.0 equivalent), Ir[(ppy)_2_(dtbbpy)]Cl (5 mol%), NaHCO_3_ (0.36 mmol, 1.8 equivalent), solvent (2.0 mL), 50°C, 30-W blue LEDs, and 12 h.

With the optimal reaction conditions in hand, the scope of anilines was explored in the next step (Figure 2). Anilines with diverse alkyl substituents on the para position could afford corresponding products in good yields (**3a**–**3e**). A strong electron-donating methoxyl group-attached product **3f** could be synthesized in a 55% yield. Unprotected hydroxyl group-containing aniline was also used as a suitable substrate, giving the product **3g** in a 41% yield. The cyclopropane unit remained intact under the standard reaction conditions as **3h** was furnished in a 43% yield. Triphenylamine could be mono-sulfonylated on the para position in this reaction system to provide **3i** in a 70% yield, probably because the electron-deficient sulfonyl group hindered the di-sulfonylation process. The protecting group of the nitrogen atom was also varied. In addition, diethyl group-attached aniline was sulfonylated to result in **3j** in a 57% yield. Both para and ortho sulfonylation (**3k** and **3l**) were observed when unsubstituted aniline was used. Sulfonylation of diamine only resulted in a 35% yield of **3m**, probably because the diamine was too reactive. In addition, electron-withdrawing group-attached aniline could not be employed in this system, probably because of the lower reactivity.

**FIGURE 3:**
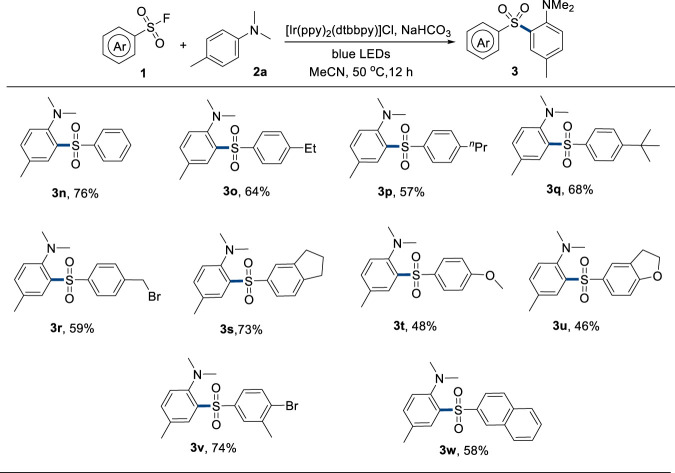
Scope of sulfonyl fluorides. Reaction conditions: **1** (0.36 mmol, 1.8 equivalent), **2a** (0.2 mmol, 1.0 equivalent), Ir[(ppy)_2_(dtbbpy)]Cl (5 mol%), NaHCO_3_ (0.36 mmol, 1.8 equiv), solvent (2.0 mL), 50°C, 30-W blue LEDs, and 12 h.

Subsequently, the scope of sulfonyl fluorides was tested (Figure 3). Para alkyl-substituted substrates provided moderate to good yields of the corresponding products (**3m**–**3p**). Bromomethyl and aryl bromide groups survived under the reaction conditions (**3q** and **3u**), which could proceed to late-stage functionalization to construct diverse and complex sulfones. Electron-donating and bicyclic sulfonyl fluorides could also generate the corresponding products in moderate to good yields (**3r**–**3t** and **3v**). However, alkyl sulfonyl fluoride could not afford the desired product.

**FIGURE 4:**
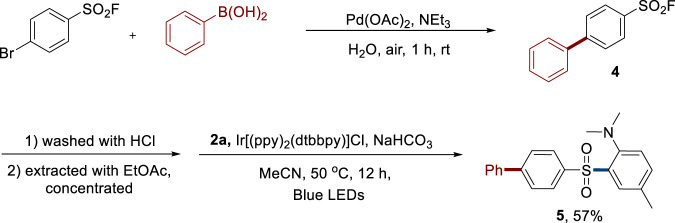
Pre-functionalization of sulfonyl fluoride and the late-stage sulfonylation.

In order to outline the advantage of our protocol, a route for late-stage sulfonylation of aniline was developed (Figure 4). First, a Suzuki–Miyaura cross-coupling reaction (Chinthakindi et al., 2016) was performed with 4-bromobenzenesulfonyl fluoride and phenylboronic acid to generate functionalized sulfonyl fluoride **4**. Then, the crude product obtained by a simple work-up was used under the standard reaction conditions. A 57% yield of functionalized product **5** was synthesized. This success highlighted that sulfonyl fluorides could be used as modifiable, stable, and atom economic sulfonyl reagents to synthesize diverse complex sulfones.

**FIGURE 5:**
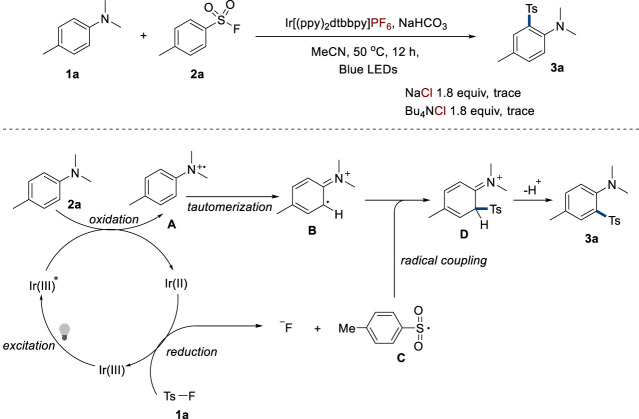
Proposed mechanism. **(A)** Radical cation intermediate. **(B)** Tautomerized intermediate. **(C)** Sulfonyl radical. **(D)** Radical coupling product.

In an attempt to gain a deep insight into the reaction mechanism, a control experiment was performed to explore the effect of the counterion (Figure 5). To investigate whether sulfonyl chloride generated from the reaction of sulfonyl fluoride and chloride anions in Ir[(ppy)_2_(dtbbpy)]Cl was the authentic substrate, NaCl or Bu_4_NCl with a combination of Ir[(ppy)_2_(dtbbpy)]PF_6_ was employed in the model reaction. However, only a trace amount of the product was detected, implying that sulfonyl fluoride was the authentic substrate. In addition, the reference also implies that harsh reaction conditions are required in the generation of sulfonyl chloride from sulfonyl fluoride (Norris et al., 1978). Based on this result and previous explorations in references (Johnson et al., 2018; Zhou et al., 2018; Lu et al., 2019; Nikl et al., 2019; Wu et al., 2019; Xu et al., 2022), a proposed mechanism is depicted in Figure 5. Aniline **2a** was oxidized by the photoexcited catalyst Ir[(ppy)_2_(dtbbpy)]Cl (Ir(III)^*^), generating radical cation **A** (Brown et al., 2015) and Ir(II) complex. This reduced iridium species possessed high reduction potential and could reduce sulfonyl fluoride to result in fluoride anion and sulfonyl radical **C** via single electron transfer (SET). After that, the radical coupling of **C** and the intermediate **B** generated from the tautomerization of **A** followed to generate the desired product **3a**.

## 3 Conclusion

In summary, we have developed a useful and simple strategy to synthesize sulfonylated anilines via visible-light-mediated reactions of sulfonyl fluorides and anilines. It has been demonstrated that sulfonyl fluorides could be used as modifiable and stable sulfonylation reagents to carry out late-stage functionalization and synthesize complex and diverse sulfones. The reaction conditions are simple and mild. In addition, the mechanism research reveals that the counterion of the photocatalyst has a crucial effect on the efficiency, which would inspire further exploration in this field.

## Data Availability

The original contributions presented in the study are included in the article/Supplementary Material; further inquiries can be directed to the corresponding author.

## References

[B1] Banks R. E. Besheesh M. K. Mohialdin-Khaffaf S. N. Sharif I. (1996). *N*-halogeno compounds. Part 18. l-Alky1-4-fluoro-l,4-diazoniabicyclo[2.2.2]octane salts: User-friendly site-selective electrophilic fluorinating agents of the *N*-fluoroammonium class. J. Chem. Soc. Perkin Trans. 1, 2069–2076. 10.1039/P19960002069

[B2] Brown T. A. Chen H. Zare R. N. (2015). Detection of the short-lived radical cation intermediate in the electrooxidation of *N,N*-dimethylaniline by mass spectrometry. Angew. Chem. Int. Ed. 54, 11183–11185. 10.1002/anie.201506316 26352029

[B3] Chinthakindi P. K. Arvidsson P. I. (2018). Sulfonyl fluorides (SFs): More than click reagents? Eur. J. Org. Chem. 2018, 3648–3666. 10.1002/ejoc.201800464

[B4] Chinthakindi P. K. Kruger H. G. Govender T. Naicker T. Arvidsson P. I. (2016). On-water synthesis of biaryl sulfonyl fluorides. J. Org. Chem. 81, 2618–2623. 10.1021/acs.joc.5b02770 26900892

[B5] Davies A. T. Curto J. M. Bagley S. W. Willis M. C. (2017). One-pot palladium-catalyzed synthesis of sulfonyl fluorides from aryl bromides. Chem. Sci. 8, 1233–1237. 10.1039/c6sc03924c 28451264 PMC5369543

[B6] Dong J. Krasnova L. Finn M. G. Sharpless K. B. (2014). Sulfur(VI) fluoride exchange (SuFEx): Another good reaction for click chemistry. Angew. Chem. Int. Ed. 53, 9430–9448. 10.1002/anie.201309399 25112519

[B7] Du X. Zhen J. S. Xu X. H. Yuan H. Li Y. H. Zheng Y. (2022). Hydrosulfonylation of alkenes with sulfonyl imines via Ir/Cu dual photoredox catalysis. Org. Lett. 24, 3944–3949. 10.1021/acs.orglett.2c01260 35617159

[B8] Durie K. Yatvin J. Kovaliov M. Crane G. H. Horn J. Averick S. (2018). SuFEx postpolymerization modification kinetics and reactivity in polymer brushes. Macromolecules 51, 297–305. 10.1021/acs.macromol.7b02372

[B9] Emmett Edward J. Willis Michael C. (2015). The development and application of sulfur dioxide surrogates in synthetic organic chemistry. Asian J. Org. Chem. 4, 602–611. 10.1002/ajoc.201500103

[B10] Ertl P. Altmann E. McKenna J. M. (2020). The most common functional groups in bioactive molecules and how their popularity has evolved over time. J. Med. Chem. 63, 8408–8418. 10.1021/acs.jmedchem.0c00754 32663408

[B11] Fadeyi O. Parikh M. D. Chen M. Z. Kyne R. E. Jr. Taylor A. P. O'Doherty I. (2016). Chemoselective preparation of clickable aryl sulfonyl fluoride monomers: A toolbox of highly functionalized intermediates for chemical biology probe synthesis. Chembiochem 17, 1925–1930. 10.1002/cbic.201600427 27504718

[B12] Gao B. Zhang L. Zheng Q. Zhou F. Klivansky L. M. Lu J. (2017). Bifluoride-catalysed sulfur(VI) fluoride exchange reaction for the synthesis of polysulfates and polysulfonates. Nat. Chem. 9, 1083–1088. 10.1038/nchem.2796 29064495 PMC5972039

[B13] Hett E. C. Xu H. Geoghegan K. F. Gopalsamy A. Kyne R. E. Jr. Menard C. A. (2015). Rational targeting of active-site tyrosine residues using sulfonyl fluoride probes. ACS Chem. Biol. 10, 1094–1098. 10.1021/cb5009475 25571984

[B14] Hyatt J. A. White A. W. (1984). Synthesis of aryl alkyl and aryl vinyl sulfones via friedel-crafts reactions of sulfonyl fluorides. Synthesis 1984, 214–217. 10.1055/s-1984-30774

[B15] Jang D. Kim J.-G. (2010). A convenient, one-pot procedure for the preparation of acyl and sulfonyl fluorides using Cl_3_CCN, Ph_3_P, and TBAF(t-BuOH)_4_ . Synlett 2010, 3049–3052. 10.1055/s-0030-1259051

[B16] Johnson T. C. Elbert B. L. Farley A. J. M. Gorman T. W. Genicot C. Lallemand B. (2018). Direct sulfonylation of anilines mediated by visible light. Chem. Sci. 9, 629–633. 10.1039/c7sc03891g 29629128 PMC5868301

[B17] Jones L. H. (2018). Emerging utility of fluorosulfate chemical probes. ACS Med. Chem. Lett. 9, 584–586. 10.1021/acsmedchemlett.8b00276 30034581 PMC6047027

[B18] Lee S. W. Woo C. W. Kim J. G. (1994). Selectivity of oxomemazine for the M_1_ muscarinic receptors. Arch. Pharm. Res. 17, 443–451. 10.1007/bf02979123 10319156

[B19] Lu F. Li J. Wang T. Li Z. Jiang M. Hu X. (2019). Electrochemical oxidative C−H sulfonylation of anilines. Asian J. Org. Chem. 8, 1838–1841. 10.1002/ajoc.201900447

[B20] Luo Y. Ding H. Zhen J. S. Du X. Xu X. H. Yuan H. (2021). Catalyst-free arylation of sulfonamides via visible light-mediated deamination. Chem. Sci. 12, 9556–9560. 10.1039/d1sc02266k 34349930 PMC8279011

[B21] Martin-Gago P. Olsen C. A. (2019). Arylfluorosulfate-based electrophiles for covalent protein labeling: A new addition to the arsenal. Angew. Chem. Int. Ed. 58, 957–966. 10.1002/anie.201806037 PMC6518939 30024079

[B22] Mortenson D. E. Brighty G. J. Plate L. Bare G. Chen W. Li S. (2018). Inverse drug discovery" strategy to identify proteins that are targeted by latent electrophiles as exemplified by aryl fluorosulfates. J. Am. Chem. Soc. 140, 200–210. 10.1021/jacs.7b08366 29265822 PMC5762408

[B23] Nikl J. Ravelli D. Schollmeyer D. Waldvogel S. R. (2019). Straightforward electrochemical sulfonylation of arenes and aniline derivatives using sodium sulfinates. ChemElectroChem 6, 4450–4455. 10.1002/celc.201901212

[B24] Norris T. (1978). The reaction of arenesulphonyl fluorides with anhydrous aluminium chloride. J. Chem. Soc. Perkin Trans. 1, 1378–1380. 10.1039/P19780001378

[B25] Qin H. L. Zheng Q. Bare G. A. Wu P. Sharpless K. B. (2016). A heck-matsuda process for the synthesis of beta-arylethenesulfonyl fluorides: Selectively addressable bis-electrophiles for SuFEx click chemistry. Angew. Chem. Int. Ed. 55, 14155–14158. 10.1002/anie.201608807 PMC5102157 27723200

[B26] Qiu G. Zhou K. Wu J. (2018). Recent advances in the sulfonylation of C-H bonds with the insertion of sulfur dioxide. Chem. Commun. 54, 12561–12569. 10.1039/c8cc07434h 30349917

[B27] Souers A. J. Leverson J. D. Boghaert E. R. Ackler S. L. Catron N. D. Chen J. (2013). ABT-199, a potent and selective BCL-2 inhibitor, achieves antitumor activity while sparing platelets. Nat. Med. 19, 202–208. 10.1038/nm.3048 23291630

[B28] Sturino C. F. O’Neill G. Lachance N. Boyd M. Berthelette C. Labelle M. (2007). Discovery of a potent and selective prostaglandin D2 receptor antagonist, [(3R)-4-(4-Chlorobenzyl)-7-fluoro-5-(methylsulfonyl)-1,2,3,4-tetrahydrocyclopenta[b]indol-3-yl]-acetic acid (MK-0524). J. Med. Chem. 50, 794–806. 10.1021/jm0603668 17300164

[B29] Sulkowski M. S. Agarwal K. Ma X. Nguyen T. T. Schiff E. R. Hann H. L. (2022). Safety and efficacy of vebicorvir administered with entecavir in treatment-naive patients with chronic hepatitis B virus infection. J. Hepatol. 77, 1265–1275. 10.1016/j.jhep.2022.05.027 35697332

[B30] Wright S. W. Hallstrom K. N. A. (2005). A convenient preparation of heteroaryl sulfonamides and sulfonyl fluorides from heteroaryl thiols. J. Org. Chem. 71, 1080–1084. 10.1021/jo052164+ 16438524

[B31] Wu Y. C. Jiang S. S. Luo S. Z. Song R. J. Li J. H. (2019). Transition-metal- and oxidant-free directed anodic C-H sulfonylation of *N,N*-disubstituted anilines with sulfinates. Chem. Commun. 55, 8995–8998. 10.1039/c9cc03789f 31290859

[B32] Xu X. H. Zhen J. S. Du X. Yuan H. Li Y. H. Chu M. H. (2022). Visible-light-mediated late-stage sulfonylation of anilines with sulfonamides. Org. Lett. 24, 853–858. 10.1021/acs.orglett.1c04144 35048703

[B33] Ye S. Li X. Xie W. Wu J. (2019). Photoinduced sulfonylation reactions through the insertion of sulfur dioxide. Eur. J. Org. Chem. 2020, 1274–1287. 10.1002/ejoc.201900396

[B34] Zhang J. Wang P. Li Y. Wu J. (2023). Asymmetric sulfonylation with sulfur dioxide surrogates: A new access to enantiomerically enriched sulfones. Chem. Commun. 59, 3821–3826. 10.1039/d2cc06339e 36880285

[B35] Zhen J. Du X. Xu X. Li Y. Yuan H. Xu D. (2022). Visible-light-mediated late-stage sulfonylation of boronic acids via N–S bond activation of sulfonamides. ACS Catal. 12, 1986–1991. 10.1021/acscatal.1c05669

[B36] Zhou K. Zhang J. Lai L. Cheng J. Sun J. Wu J. (2018). C-H bond sulfonylation of anilines with the insertion of sulfur dioxide under metal-free conditions. Chem. Commun. 54, 7459–7462. 10.1039/c8cc03465f 29911698

