# Supplementary material for: Visible-light-mediated sulfonylation of anilines with sulfonyl fluorides
Source: Front Chem. 2023 Aug 25;11:1267223. doi: 10.3389/fchem.2023.1267223 (PMC10485258; doi:10.3389/fchem.2023.1267223)
Supplement: Supplementary file 1 [file DataSheet1.PDF]

Visible-light-mediated sulfonylation of anilines with sulfonyl fluorides

Supplementary Material

1. General information……………………………………………S2

2. Experiment procedures…………………………………………S3

3. Characterization Data for Substrates……………………………S5

4. References…………………………………………………………S12

5. ^{1}H NMR and ^{13}C NMR spectra…………………………………..S13

1. General information

All experiments were performed using standard Schlenk techniques under nitrogen atmosphere. All anhydrous solvents, commercial anilines and sulfonyl fluorides were purchased from Adamas-Beta Co. or Bidepharm Co. and used as received. All photoredox experiments were conducted under a 455 nm strip from Xuzhou Aijia Electronic Technology Co., Ltd, as shown in figure S1 and figure S2. And the reaction vessel placed ~1 cm from the lamp. Reactions remained at ~50 °C. All products were identified using NMR analysis and comparison with authentic samples. ^{1}H NMR and ^{13}C NMR spectra were recorded in $CDCl_3$ on Bruker spectrometers at 400 MHz. All shifts are reported in parts per million (ppm) relative to residual $CHCl_3$ peak (7.26 and 77.0 ppm, ^{1}H NMR and ^{13}C NMR, respectively). Abbreviations are: s, singlet; d, doublet; t, triplet; q, quartet; brs, broad singlet. High Resolution Mass Spectra were obtained on a Thermofisher LTQ Orbitrap Elite (ESI). All flash chromatography was perform using silica gel, 300-400 mesh. TLC analysis was carried out on glass plates coated with silica gel 60 F254, 0.2 mm thickness. The plates were visualized using a 254 nm ultraviolet lamp or iodine cylinder.

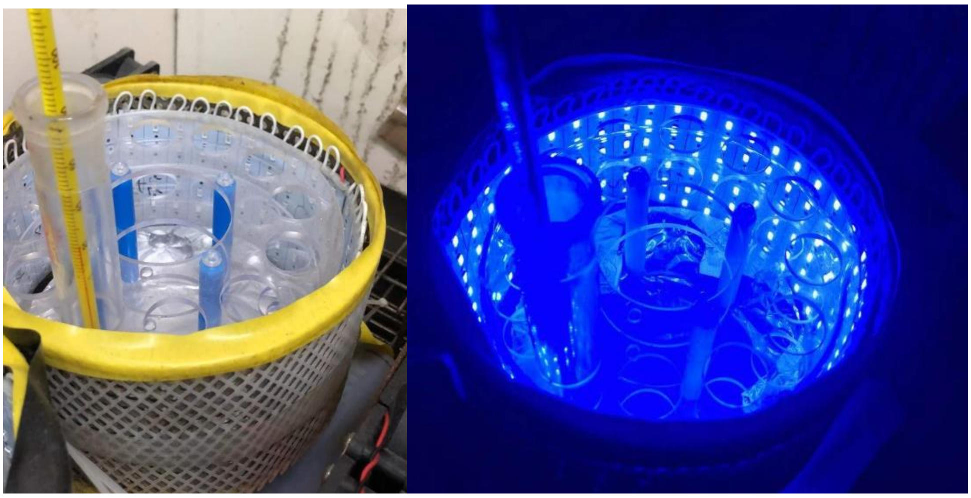

Figure S1

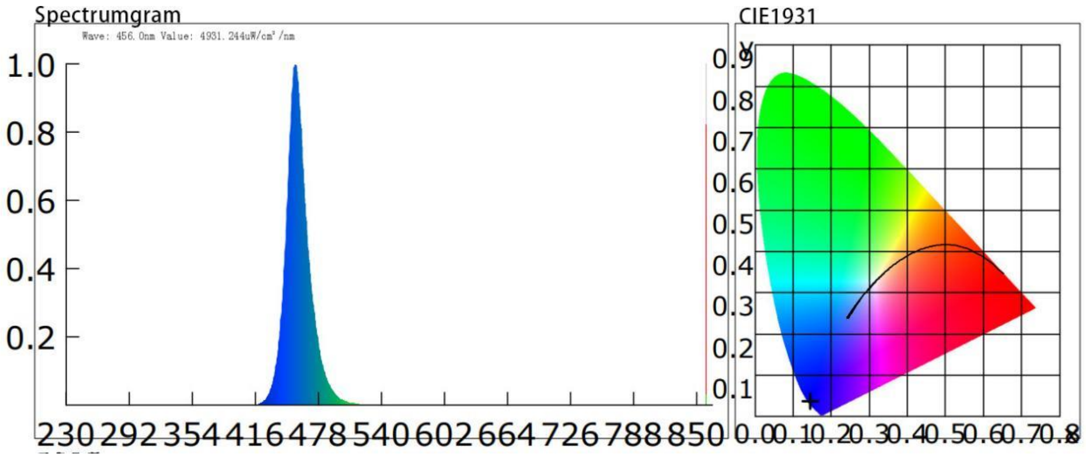

Figure S2

2. Experiment procedures

2.1 Preparation of Sulfonyl Fluorides (Cl-F exchanging)

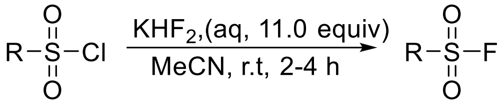

Sulfonyl chloride (5 mmol, 1.0 equiv) was dissolved in MeCN (10 mL), and aq. KHF_2 (10 mL, 11.0 equiv) was added. The suspension was stirred for 2-4 hours at room temperature. Then the reaction mixture was diluted with H_2O (5 mL) and extracted with EtOAc (3 x 5 mL). The combined organic layer was dried over Na_2SO_4, and filtrate was concentrated in vacuo to directly give the desired sulfonyl fluoride **1**.

2.2 Preparation of Aryl amines

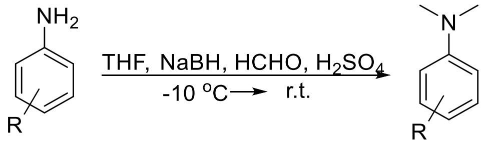

An oven-dried round-bottomed flask (250 mL) equipped with a stir bar was charged with concentrated sulfuric acid (140 mmol, 14 equiv) and formaldehyde (70 mmol, 7.0 equiv). To this flask was added dropwise the mixture of aryl amine (10 mmol, 1.0 equiv), $NaBH_4$ (70 mmol, 7.0 equiv), and THF (70 mL) with vigorous stirring at -10 °C. After stirred overnight at room temperature, the reaction mixture was washed with dilute sodium hydroxide, brine, concentrated, dried, and purified by column chromatography to get the amine **2**.

2.3 General Procedure A: Substrate Scope of Sulfonyl Fluorides

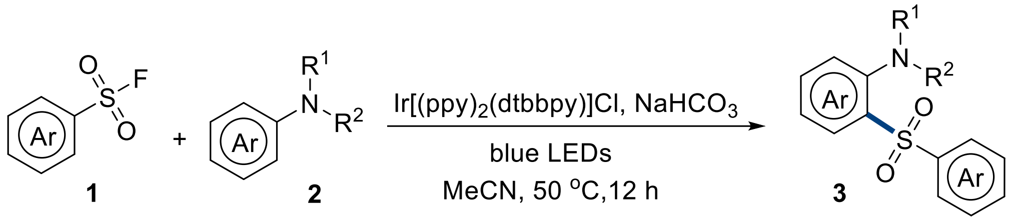

An oven-dried Schlenk tube equipped with a stir bar was charged with sulfonyl fluoride **1** (0.36 mmol, 1.8 equiv), aryl amine **2** (0.2 mmol, 1.0 equiv), $NaHCO_3$ (0.36 mmol, 1.8 equiv) and Ir[$(ppy)_2$dtbpy]Cl (5 mol%). Then the tube was sealed with a cap, evacuated and backfilled with nitrogen for 3 times. Subsequently, the solvent CH_3CN (2 mL) was added. The tube was under irradiation by 30 W blue LEDs (455 nm, distance: 1 cm, the reaction temperature reached 50 °C) for 12 hours. Then the mixture was washed with H_2O (1 x 2 mL), diluted with EtOAc (1 x 4 mL), and concentrated. The crude product was purified by flash chromatography (EtOAc/PE: 1/4, unless otherwise noted) to give analytically pure product **3**.

2.4 Cross-coupling

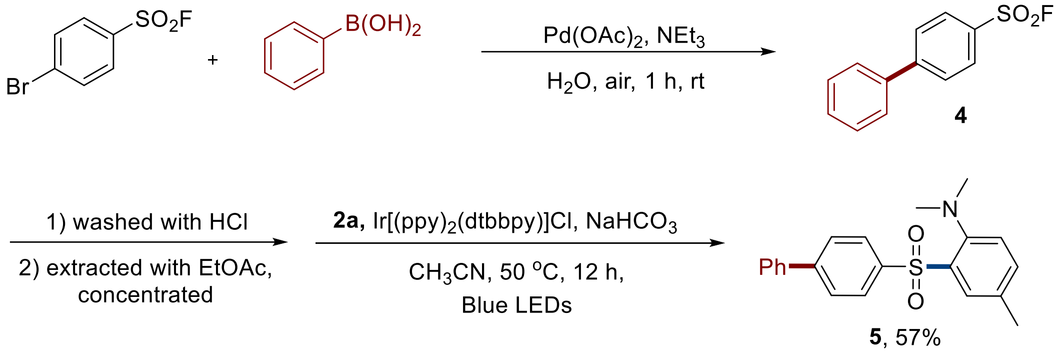

An oven-dried Schlenk tube equipped with a stir bar was charged with 4-bromobenzenesulfonyl fluoride (0.3 mmol, 1.5 equiv), phenylboronic acid (0.45 mmol, 2.25 equiv), NEt_3 (0.9 mmol, 4.5 equiv), palladium (II) acetate (1 mol%) and H_2O (1 mL). The suspension was stirred under air at room temperature for 1 hour. After that, the suspension was washed with aq. HCl (1 mL, 1 M), and extracted with EtOAc (2 x 1 mL). The organic layer was placed in a new oven-dried Schlenk tube, and concentrated. To this tube was added $NaHCO_3$ (3.6 mmol, 1.8 equiv) and Ir[$(ppy)_2$dtbbpy]Cl (5 mol%). Then the tube was sealed with a cap, evacuated and backfilled with nitrogen for 3 times. Subsequently, aryl amine **2a** (0.2 mmol, 1.0 equiv) and the solvent CH_3CN (2 mL) was added. The tube was under irradiation of 30 W blue

LEDs (455 nm, distance: 1 cm, the reaction temperature reached 50 °C) for 12 hours. Then the mixture was washed with H_2O (1 x 2 mL), diluted with EtOAc (1 x 4 mL), and concentrated. The crude product was purified by flash chromatography (EtOAc/PE: 1/4, unless otherwise noted) to give analytically pure product **3**.

3. Characterization Data for Substrates

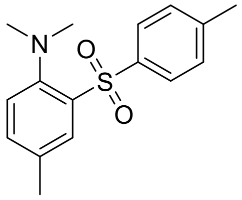

3a[1], **^{1}H NMR (400 MHz, Chloroform-*d*)** δ 8.04 (s, 1H), 7.77 (d, *J* = 8.2 Hz, 2H), 7.34 (dd, *J* = 8.1, 2.1 Hz, 1H), 7.22 (d, *J* = 8.0 Hz, 2H), 7.15 (d, *J* = 8.1 Hz, 1H), 2.40 (s, 3H), 2.38 (s, 3H), 2.37 (s, 6H). **^{13}C NMR (101 MHz, Chloroform-*d*)** δ 151.2, 143.1, 139.5, 137.9, 135.3, 135.2, 129.7, 128.6, 128.2, 124.3, 45.4, 21.6, 20.9.

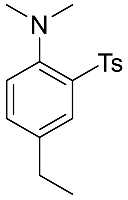

3b, ^{1}H NMR (400 MHz, Chloroform-d) δ 8.11 – 8.02 (m, 1H), 7.78 (d, J = 8.1 Hz, 2H), 7.37 (dd, J = 8.1, 1.6 Hz, 1H), 7.22 (d, J = 8.0 Hz, 2H), 7.18 (d, J = 8.1 Hz, 1H), 2.70 (q, J = 7.6 Hz, 2H), 2.38 (d, J = 5.3 Hz, 9H), 1.29 – 1.25 (t, 3H). **^{13}C NMR (101 MHz, Chloroform-*d*)** δ 151.33, 143.02, 141.55, 139.54, 137.99, 134.06, 128.61, 128.20, 124.31, 45.37, 28.37, 21.56, 15.42. **HRMS** (ESI/[M+H]$^+$) calcd for $C_{17}H_{22}NO_2S$: 304.1366, found: 304.1360.

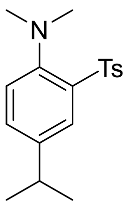

3c, ^{1}H NMR (400 MHz, Chloroform-d) δ 8.09 (d, J = 2.0 Hz, 1H), 7.78 (d, J = 8.2 Hz, 2H), 7.40 (dd, J = 8.2, 2.0 Hz, 1H), 7.21 (dd, J = 16.6, 8.1 Hz, 3H), 2.98 (p, J = 6.9 Hz, 1H), 2.38 (d, J = 5.3 Hz, 9H), 1.28 (d, J = 6.9 Hz, 6H). **^{13}C NMR (101 MHz, Chloroform-*d*)** δ 151.36, 146.19, 143.01, 139.55, 137.98, 132.62, 128.61, 128.20,

127.26, 124.33, 45.35, 33.78, 23.87, 21.56. **HRMS** (ESI/[M+H]$^+$) calcd for $C_{18}H_{24}NO_2S$: 318.1522, found: 318.1517.

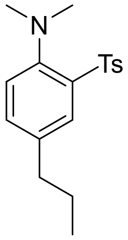

3d, ^{1}H NMR (400 MHz, Chloroform-*d*) δ 8.04 (s, 1H), 7.77 (d, *J* = 8.3 Hz, 3H), 7.35 (dd, *J* = 8.1, 2.1 Hz, 1H), 7.22 (d, *J* = 8.0 Hz, 2H), 7.17 (d, *J* = 8.1 Hz, 1H), 2.64 (t, *J* = 7.7 Hz, 2H), 2.38 (d, *J* = 3.8 Hz, 9H), 1.67 (q, *J* = 7.5 Hz, 2H), 0.96 (t, *J* = 7.3 Hz, 3H).**^{13}C NMR (101 MHz, Chloroform-*d*)** δ 151.35, 143.01, 140.04, 139.55, 137.88, 134.62, 129.17, 128.61, 128.18, 124.19, 45.38, 37.41, 24.42, 21.55, 13.75. **HRMS** (ESI/[M+H]$^+$) calcd for $C_{18}H_{24}NO_2S$: 318.1522, found: 318.1518.

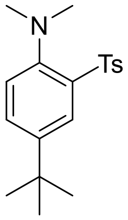

3e, ^{1}H NMR (400 MHz, Chloroform-*d*) δ 8.24 (d, *J* = 2.4 Hz, 1H), 7.78 (d, *J* = 8.1 Hz, 2H), 7.56 (dd, *J* = 8.4, 2.4 Hz, 1H), 7.21 (dd, *J* = 16.1, 8.2 Hz, 3H), 2.38 (d, *J* = 3.4 Hz, 9H), 1.36 (s, 9H). **^{13}C NMR (101 MHz, Chloroform-*d*)** δ 151.0, 148.6, 143.0, 139.6, 137.6, 131.6, 128.6, 128.2, 126.2, 124.0, 45.3, 34.9, 31.3, 21.6. **HRMS** (ESI/[M+H]$^+$) calcd for $C_{19}H_{26}NO_2S$: 332.1679, found: 332.1670.

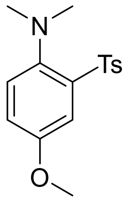

3f[2], ^{1}H NMR (400 MHz, Chloroform-*d*) δ 7.78 (d, *J* = 8.0 Hz, 2H), 7.75 (d, *J* = 3.1 Hz, 1H), 7.22 (dd, *J* = 13.8, 8.4 Hz, 3H), 7.08 (dd, *J* = 8.7, 3.0 Hz, 1H), 3.87 (s, 3H), 2.39 (s, 3H), 2.32 (s, 6H). **^{13}C NMR (101 MHz, Chloroform-*d*)** δ 156.8, 146.4, 143.2, 139.4, 139.3, 128.6, 128.3, 125.7, 121.3, 113.0, 55.9, 45.4, 21.6.

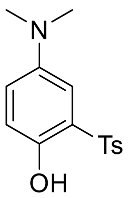

3g[3]**, ^{1}H NMR (400 MHz, Chloroform-d)** δ 8.55 (s, 1H), 7.82 (d, J = 8.0 Hz, 2H), 7.30 (d, J = 8.0 Hz, 2H), 6.91 (m, 3H), 2.86 (s, 6H), 2.39 (s, 3H). **^{13}C NMR (101 MHz, DMSO-*d*$_6$)** δ 142.35, 140.39, 139.78, 134.21, 125.22, 122.04, 118.82, 117.21, 114.94, 106.29, 36.44, 16.85.

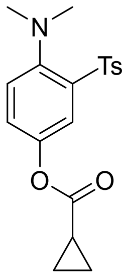

3h, ^{1}H NMR (400 MHz, Chloroform-*d*) δ 7.96 (d, *J* = 2.7 Hz, 1H), 7.78 (d, *J* = 8.0 Hz, 2H), 7.30 (dd, *J* = 8.6, 2.7 Hz, 1H), 7.24 (dd, *J* = 8.3, 5.3 Hz, 3H), 2.39 (d, *J* = 1.8 Hz, 9H), 1.85 (td, *J* = 8.1, 4.0 Hz, 1H), 1.21 – 1.16 (m, 2H), 1.07 (dt, *J* = 8.1, 3.5 Hz, 2H). **^{13}C NMR (101 MHz, Chloroform-*d*)** δ 173.3, 151.0, 147.3, 143.5, 139.3, 138.8, 128.7, 128.4, 128.0, 125.3, 122.9, 45.5, 21.6, 12.9, 9.5. **HRMS** (ESI/[M+H]$^+$) calcd for $C_{19}H_{22}NO_4S$: 360.1264, found: 360.1261.

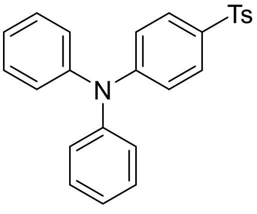

3i[4]**, ^{1}H NMR (400 MHz, Chloroform-*d*)** δ 7.91 – 7.75 (m, 2H), 7.73 – 7.61 (m, 2H), 7.32 – 7.27 (m, 5H), 7.15 – 7.09 (m, 5H), 7.00 – 6.89 (m, 2H), 2.39 (s, 3H). **^{13}C NMR (101 MHz, Chloroform-*d*)** δ 152.0, 146.1, 143.6, 139.6, 132.2, 129.8, 129.8, 128.9, 127.4, 126.2, 125.1, 119.6, 21.6.

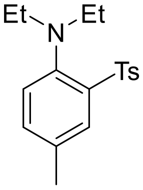

3j, ^{1}H NMR (400 MHz, Chloroform-*d*) δ 8.13 (d, *J* = 2.1 Hz, 1H), 7.75 (d, *J* = 8.2 Hz, 2H), 7.33 (dd, *J* = 8.1, 2.1 Hz, 1H), 7.20 (s, 2H), 7.06 (d, *J* = 8.0 Hz, 1H), 2.84 (q, *J* = 7.1 Hz, 4H), 2.42 (s, 3H), 2.39 (s, 3H), 0.55 (t, *J* = 7.1 Hz, 6H). **^{13}C NMR (101 MHz, Chloroform-*d*)** δ 148.5, 143.2, 139.4, 138.1, 134.6, 134.5, 130.5, 128.7, 128.6, 125.6, 48.2, 21.5, 20.9, 11.1. **HRMS** (ESI/[M+H]$^{+}$) calcd for $C_{18}H_{24}NO_2S$: 318.1522, found: 318.1517.

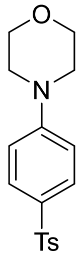

3k[5], ^{1}H NMR (400 MHz, Chloroform-d) δ 7.78 (d, J = 8.5 Hz, 4H), 7.26 (d, J = 7.6 Hz, 2H), 6.87 (d, J = 8.7 Hz, 2H), 3.85 – 3.78 (m, 4H), 3.30 – 3.21 (m, 4H), 2.37 (s, 3H). **^{13}C NMR (101 MHz, Chloroform-*d*)** δ 153.99, 143.42, 139.87, 130.51, 129.75, 129.30, 127.19, 113.84, 66.47, 47.42, 21.51.

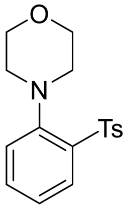

3l, ^{1}H NMR (400 MHz, Chloroform-d) δ 8.30 (d, J = 8.8 Hz, 1H), 7.76 (d, J = 8.2 Hz, 2H), 7.61 (t, J = 7.7 Hz, 1H), 7.38 (t, J = 7.6 Hz, 1H), 7.27 (dd, J = 16.2, 8.0 Hz, 3H), 3.67 – 3.58 (t, 4H), 2.79 – 2.68 (t, 4H), 2.39 (s, 3H). **^{13}C NMR (101 MHz, Chloroform-*d*)** δ 152.30, 143.34, 139.67, 137.46, 134.88, 130.61, 128.93, 127.60, 125.65, 124.36, 66.66, 53.53, 21.56. **HRMS** (ESI/[M+H]$^{+}$) calcd for $C_{17}H_{20}NO_3S$: 318.1158, found 318.1151.

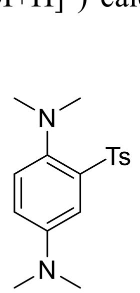

3m[6], ^{1}H NMR (400 MHz, Chloroform-*d*) δ 8.13 (d, *J* = 8.0 Hz, 1H), 7.74 (d, *J* = 8.2

Hz, 2H), 7.49 (t, *J* = 8.2 Hz, 1H), 7.26 – 7.13 (m, 3H), 2.93 (t, *J* = 6.1 Hz, 4H), 2.40 (s, 3H), 1.81 (p, *J* = 3.3 Hz, 4H). **^{13}C NMR (101 MHz, Chloroform-*d*)** δ 150.47, 143.10, 139.62, 136.23, 134.32, 130.29, 128.82, 127.81, 123.30, 123.03, 53.92, 24.88, 21.57.

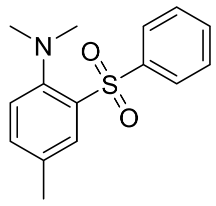

3n[1], **^{1}H NMR (400 MHz, Chloroform-*d*)** δ 8.06 (d, *J* = 2.0 Hz, 1H), 7.88 (d, *J* = 7.3 Hz, 2H), 7.55 – 7.48 (m, 1H), 7.43 (t, *J* = 7.6 Hz, 2H), 7.36 (dd, *J* = 8.1, 2.1 Hz, 1H), 7.16 (d, *J* = 8.0 Hz, 1H), 2.41 (s, 3H), 2.34 (s, 6H). **^{13}C NMR (101 MHz, Chloroform-*d*)** δ 151.2, 142.5, 137.7, 135.5, 135.3, 132.3, 129.7, 128.0, 128.0, 124.3, 45.3, 21.0.

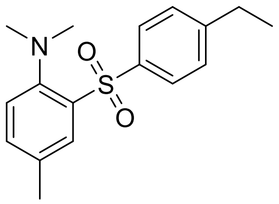

3o, ^{1}H NMR (400 MHz, Chloroform-*d*) δ 8.04 (s, 1H), 7.80 (d, *J* = 8.1 Hz, 2H), 7.34 (dd, *J* = 8.1, 2.1 Hz, 1H), 7.24 (s, 2H), 7.15 (d, *J* = 8.1 Hz, 1H), 2.68 (q, *J* = 7.6 Hz, 2H), 2.40 (s, 3H), 2.36 (s, 6H), 1.21 (t, *J* = 7.6 Hz, 3H). **^{13}C NMR (101 MHz, Chloroform-*d*)** δ 151.2, 149.3, 139.7, 138.0, 135.3, 129.7, 128.3, 127.5, 124.3, 45.3, 28.9, 20.9, 15.3. **HRMS** (ESI/$[M+H]^+$) calcd for $C_{17}H_{22}NO_2S$: 304.1366, found 304.1362.

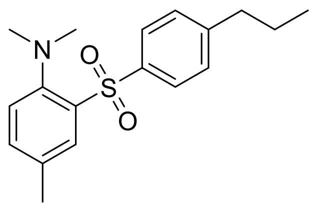

3p, ^{1}H NMR (400 MHz, Chloroform-*d*) δ 8.05 (d, *J* = 2.0 Hz, 1H), 7.79 (d, *J* = 8.4 Hz, 2H), 7.34 (dd, *J* = 8.1, 2.1 Hz, 1H), 7.22 (d, *J* = 8.1 Hz, 2H), 7.15 (d, *J* = 8.0 Hz, 1H), 2.62 (t, *J* = 7.6 Hz, 2H), 2.40 (s, 3H), 2.35 (s, 6H), 1.62 (q, *J* = 7.4 Hz, 2H), 0.89 (d, *J* = 14.7 Hz, 3H). **^{13}C NMR (101 MHz, Chloroform-*d*)** δ 151.2, 147.7, 139.7, 138.0, 135.3, 129.7, 128.2, 128.1, 124.3, 45.3, 37.8, 24.3, 20.9, 13.6. **HRMS** (ESI/$[M+H]^+$) calcd for $C_{18}H_{24}NO_2S$: 318.1522, found 318.1517.

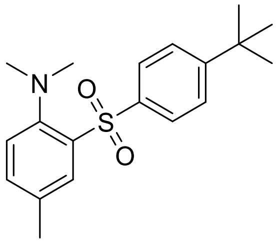

3q[1], **^{1}H NMR (400 MHz, Chloroform-d)** δ 8.05 (d, J = 2.0 Hz, 1H), 7.83 – 7.78 (m, 2H), 7.46 – 7.41 (m, 2H), 7.34 (dd, J = 8.0, 2.1 Hz, 1H), 7.15 (d, J = 8.0 Hz, 1H), 2.40 (s, 3H), 2.35 (s, 6H), 1.30 (s, 9H). **^{13}C NMR (101 MHz, Chloroform-*d*)** δ 156.1, 151.2, 139.4, 138.0, 135.3, 135.2, 129.7, 128.0, 124.9, 124.3, 45.3, 35.1, 31.1, 20.9.

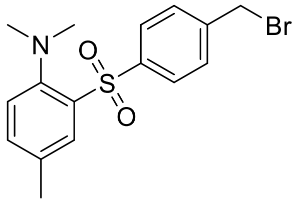

3r, ^{1}H NMR (400 MHz, Chloroform-*d*) δ 7.91 – 7.86 (m, 2H), 7.43 (d, *J* = 8.1 Hz, 2H), 7.12 – 7.03 (m, 2H), 6.85 (d, *J* = 2.0 Hz, 1H), 4.16 (s, 2H), 2.60 (s, 6H), 2.25 (s, 3H). **^{13}C NMR (101 MHz, Chloroform-d)** δ 151.14, 134.17, 131.38, 130.26, 128.52, 128.39, 120.41, 45.41, 36.99, 20.78. **HRMS** (ESI/[M+H]$^+$) calcd for $C_{16}H_{19}BrNO_2S$: 368.0314, found 368.0310.

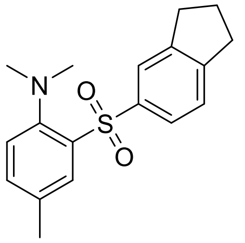

3s, ^{1}H NMR (400 MHz, Chloroform-*d*) δ 8.04 (d, *J* = 2.1 Hz, 1H), 7.75 – 7.65 (m, 2H), 7.34 (dd, *J* = 8.1, 2.1 Hz, 1H), 7.25 (d, *J* = 7.9 Hz, 1H), 7.16 (d, *J* = 8.1 Hz, 1H), 2.91 (td, *J* = 7.5, 4.0 Hz, 4H), 2.39 (d, *J* = 5.7 Hz, 9H), 2.09 (p, *J* = 7.5 Hz, 2H). **^{13}C NMR (101 MHz, Chloroform-*d*)** δ 151.3, 149.4, 144.3, 140.2, 138.1, 135.2, 135.1, 129.7, 126.6, 124.3, 124.0, 123.7, 45.4, 32.9, 32.6, 25.4, 21.0. **HRMS** (ESI/[M+H]$^+$) calcd for $C_{18}H_{22}NO_2S$: 316.1366, found 316.1361.

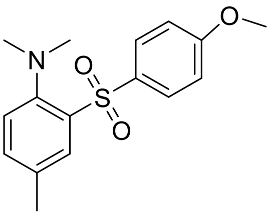

3t[1]**, ^{1}H NMR (400 MHz, Chloroform-*d*)** δ 8.03 (d, *J* = 2.1 Hz, 1H), 7.85 (d, *J* = 8.8 Hz, 2H), 7.33 (dd, *J* = 8.1, 2.1 Hz, 1H), 7.15 (d, *J* = 8.1 Hz, 1H), 6.90 (d, *J* = 8.9 Hz, 2H), 3.84 (s, 3H), 2.40 (d, *J* = 1.7 Hz, 9H). **^{13}C NMR (101 MHz, Chloroform-*d*)** δ 162.8, 151.1, 138.1, 135.2, 135.1, 134.1, 130.5, 129.6, 124.2, 113.2, 55.6, 45.5, 20.9.

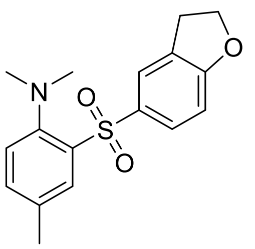

3u, ^{1}H NMR (400 MHz, Chloroform-*d*) δ 8.02 (s, 1H), 7.74 (dd, *J* = 11.0, 2.7 Hz, 2H), 7.34 (d, *J* = 8.1 Hz, 1H), 7.17 (d, *J* = 8.1 Hz, 1H), 6.77 (d, *J* = 8.3 Hz, 1H), 4.63 (t, *J* = 8.8 Hz, 2H), 3.21 (t, *J* = 8.8 Hz, 2H), 2.43 (s, 6H), 2.40 (s, 3H). **^{13}C NMR (101 MHz, Chloroform-*d*)** δ 163.7, 151.1, 138.3, 135.2, 135.1, 133.9, 130.2, 129.6, 127.1, 125.6, 124.2, 108.5, 72.2, 45.6, 29.0, 21.0. **HRMS** (ESI/[M+H]$^{+}$) calcd for $C_{17}H_{20}NO_3S$: 318.1158, found 318.1154.

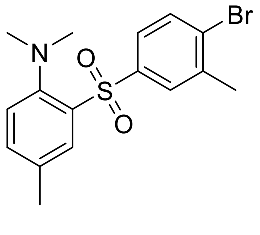

3v, ^{1}H NMR (400 MHz, Chloroform-*d*) δ 8.02 (d, *J* = 2.0 Hz, 1H), 7.75 (d, *J* = 1.8 Hz, 1H), 7.59 (d, *J* = 2.4 Hz, 2H), 7.36 (d, *J* = 2.1 Hz, 1H), 7.17 (d, *J* = 8.1 Hz, 1H), 2.42 (d, *J* = 4.1 Hz, 6H), 2.39 (s, 6H).**^{13}C NMR (101 MHz, Chloroform-*d*)** δ 151.2, 141.5, 138.2, 137.3, 135.6, 135.5, 132.0, 130.0, 129.8, 129.8, 127.0, 124.4, 45.4, 22.9, 21.0. **HRMS** (ESI/[M+H]$^{+}$) calcd for $C_{16}H_{19}BrNO_2S$: 368.0314, found 368.0311.

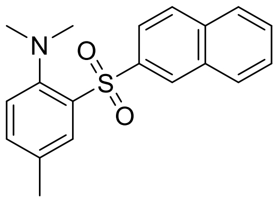

3w[1], **^{1}H NMR (400 MHz, Chloroform-*d*)** δ 8.48 (s, 1H), 8.12 (d, *J* = 2.1 Hz, 1H), 7.93 (dd, *J* = 7.8, 1.5 Hz, 1H), 7.89 – 7.83 (m, 3H), 7.62 – 7.53 (m, 2H), 7.35 (dd, *J* = 8.1, 2.1 Hz, 1H), 7.14 (d, *J* = 8.0 Hz, 1H), 2.43 (s, 3H), 2.32 (s, 6H). **^{13}C NMR (101 MHz, Chloroform-*d*)** δ 151.2, 139.5, 137.8, 135.5, 135.4, 134.8, 132.0, 129.8, 129.4, 129.2, 128.6, 127.9, 127.8, 127.1, 124.3, 123.7, 45.4, 21.0.

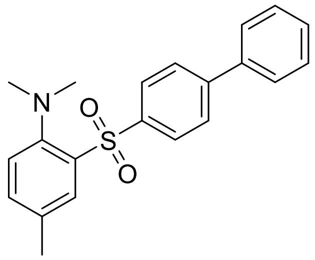

5, ^{1}H NMR (400 MHz, Chloroform-*d*) δ 8.08 (d, *J* = 2.1 Hz, 1H), 7.95 (d, *J* = 8.4 Hz, 2H), 7.65 (d, *J* = 8.4 Hz, 2H), 7.58 (d, *J* = 7.4 Hz, 2H), 7.45 (t, *J* = 7.6 Hz, 2H), 7.41 – 7.35 (m, 2H), 7.17 (d, *J* = 8.1 Hz, 1H), 2.42 (s, 3H), 2.39 (s, 6H). **^{13}C NMR (101 MHz, Chloroform-*d*)** δ 151.2, 145.2, 141.1, 139.5, 137.8, 135.5, 135.4, 129.8, 129.0, 128.7, 128.4, 127.3, 126.6, 124.4, 45.4, 21.0. **HRMS** (ESI/[M+H]$^{+}$) calcd for $C_{21}H_{22}NO_2S$: 352.1366, found 352.1363.

4. Reference

[1] Lu, F., Li, J., Wang, T., Li, Z., Jiang, M., and Hu, X. et al. (2019). Electrochemical Oxidative C−H Sulfonylation of Anilines. *Asian J. Org. Chem*. 8, 1838-1841.

[2] Wu, Y. C., Jiang, S. S., Luo, S. Z., Song, R. J., and Li, J. H. (2019). Transition-metal- and oxidant-free directed anodic C-H sulfonylation of *N,N*-disubstituted anilines with sulfinates. *Chem. Commun*. 55, 8995-8998.

[3] Torti, E., Giustina, G. D., Protti, S., Merli, D., Brusatinb, G., Fagnoni, M. (2015). Aryl tosylates as non-ionic photoacid generators (PAGs): photochemistry and applications in cationic photopolymerizations. *RSC Adv.* 5, 33239–33248.

[4] Luo, Y., Ding, H., Zhen, J.-S., Du, X., Xu, X.-H.,Yuan, H. et al. (2021). Catalyst-Free Arylation of Sulfonamides via Visible Light-Mediated Deamination. *Chem. Sci*. 12, 9556−9560.

[5] Chen, Y., Wills, M. (2017). Copper(I)-catalyzed sulfonylative Suzuki-Miyaura cross-coupling.*Chem. Sci.* 8, 3249–3253.

[6] Nematollahi, D., Hosseinzadeh, S., Dadpou, B. (2015). Comproportionation and Michael addition reactions of electrochemically generated *N*,*N*,*N′*,*N′*-tetramethyl-1,4-phenylenediamine dication. Synthesis of new unsymmetrical aryl sulfones containing *N*,*N*,*N′*,*N′*-tetramethyl-1,4-phenylenediamine moiety. *J. Electroanal. Chem.* 759, 144–152.

5. ^{1}H NMR and ^{13}C NMR spectra

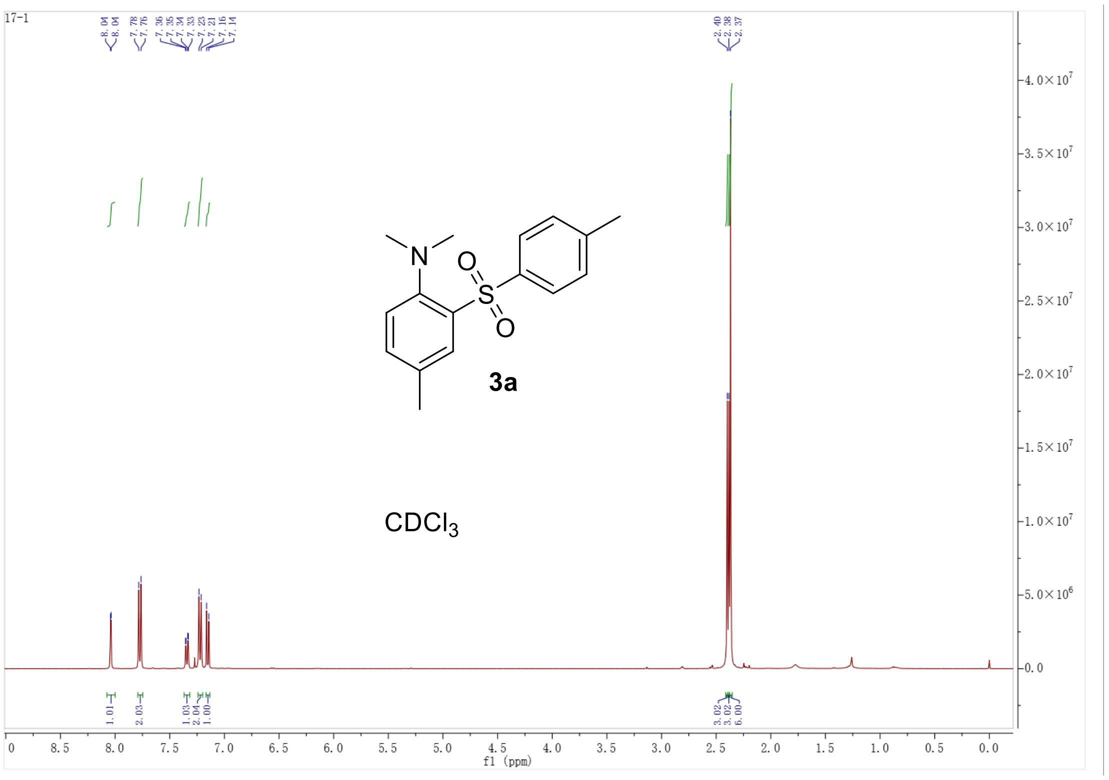

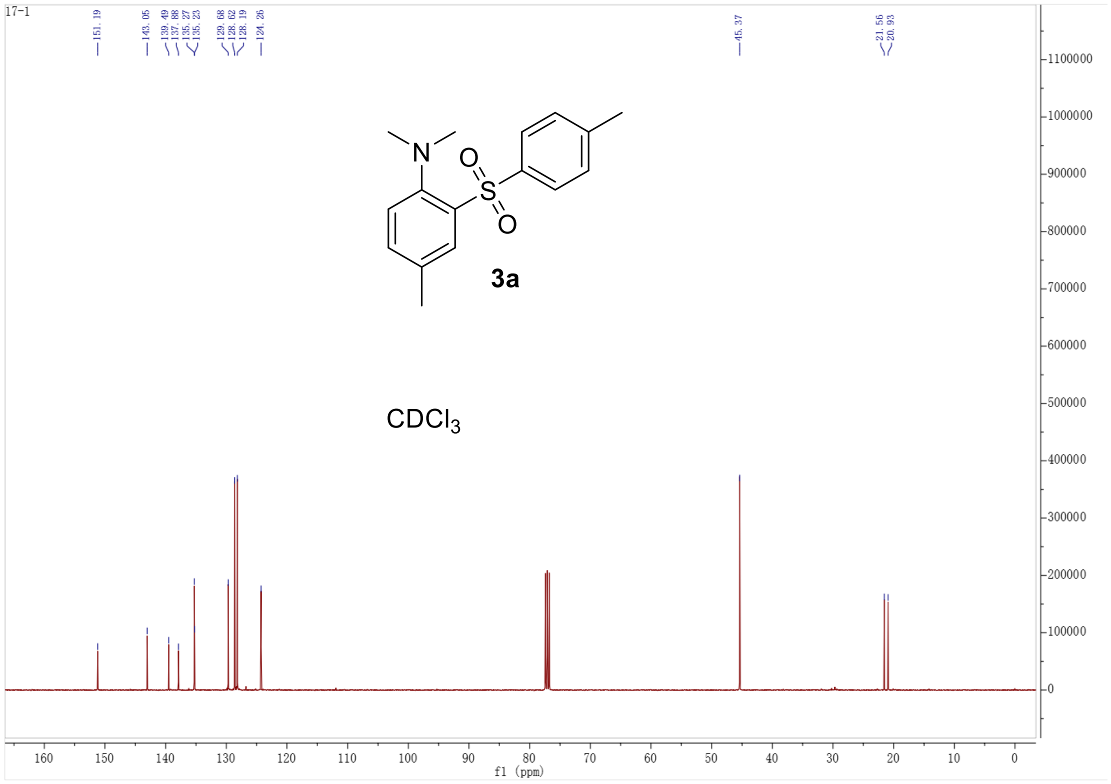

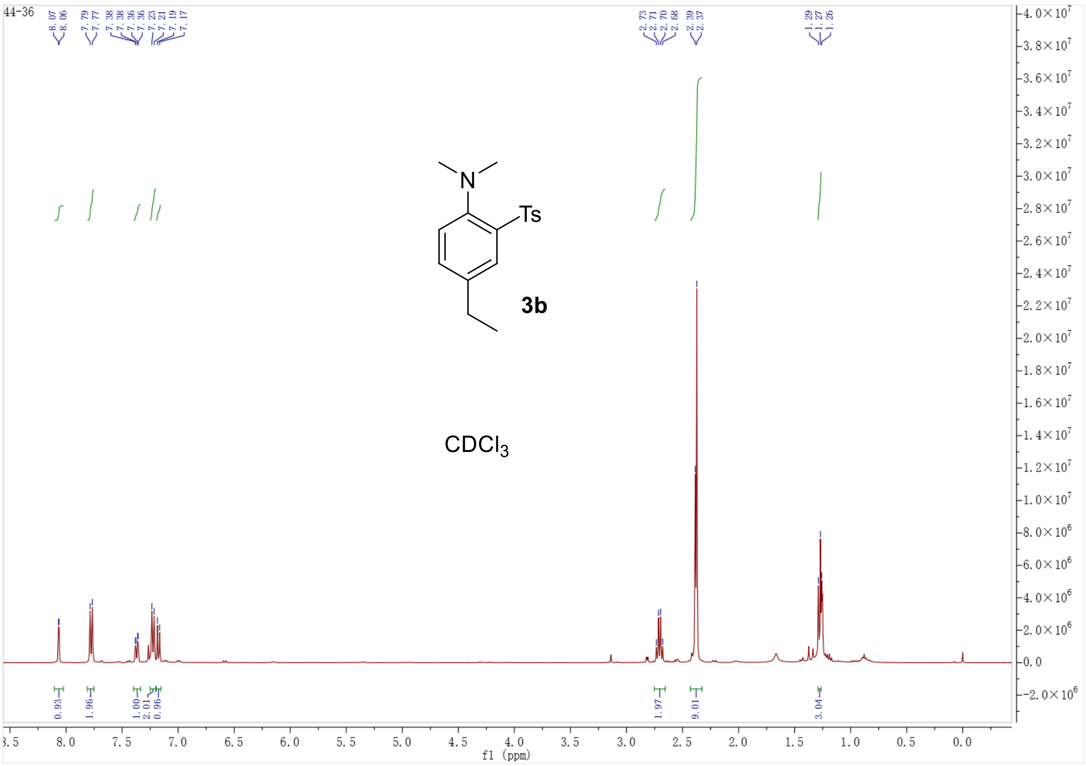
44-36
8.07
8.06
7.79
7.77
7.38
7.38
7.36
7.36
7.23
7.21
7.19
7.17
2.73
2.71
2.70
2.68
2.39
2.37
1.29
1.27
1.26
Ts
N
3b
CDCl3
0.93
1.96
1.00
2.01
0.96
1.97
9.01
3.04
f1 (ppm)

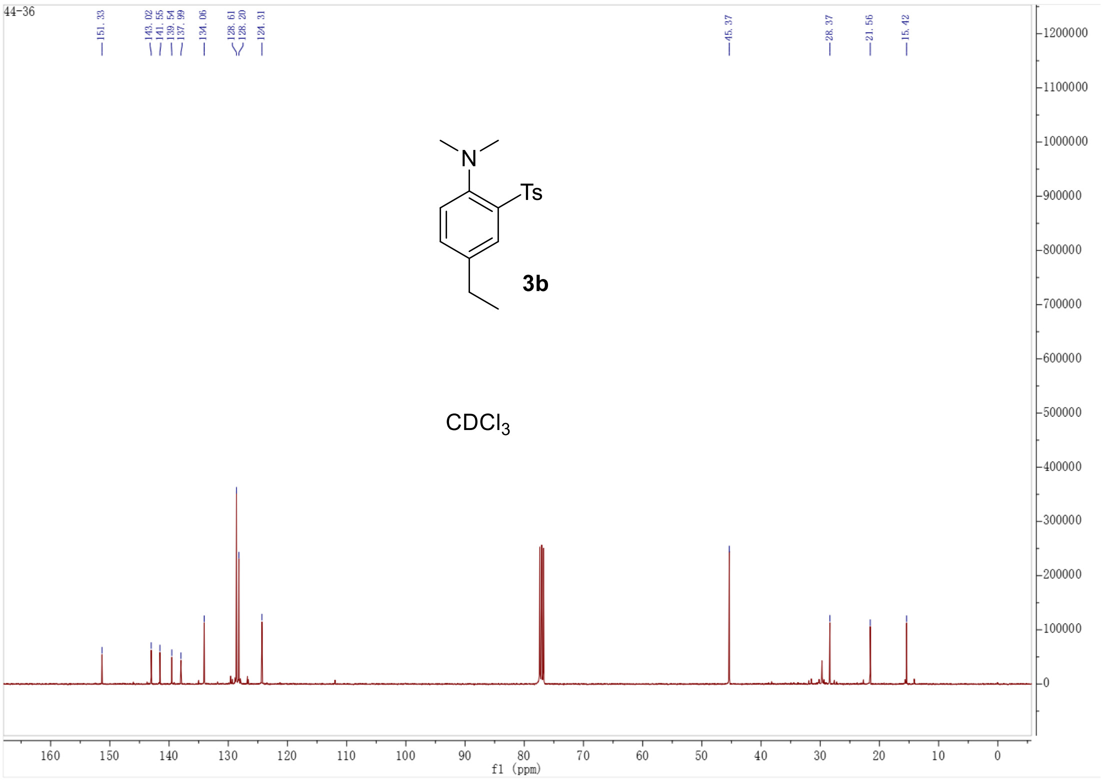
44-36
151.33
143.02
141.55
139.54
137.99
134.06
128.61
128.20
124.31
45.37
28.37
21.56
15.42
Ts
N
3b
CDCl3
f1 (ppm)

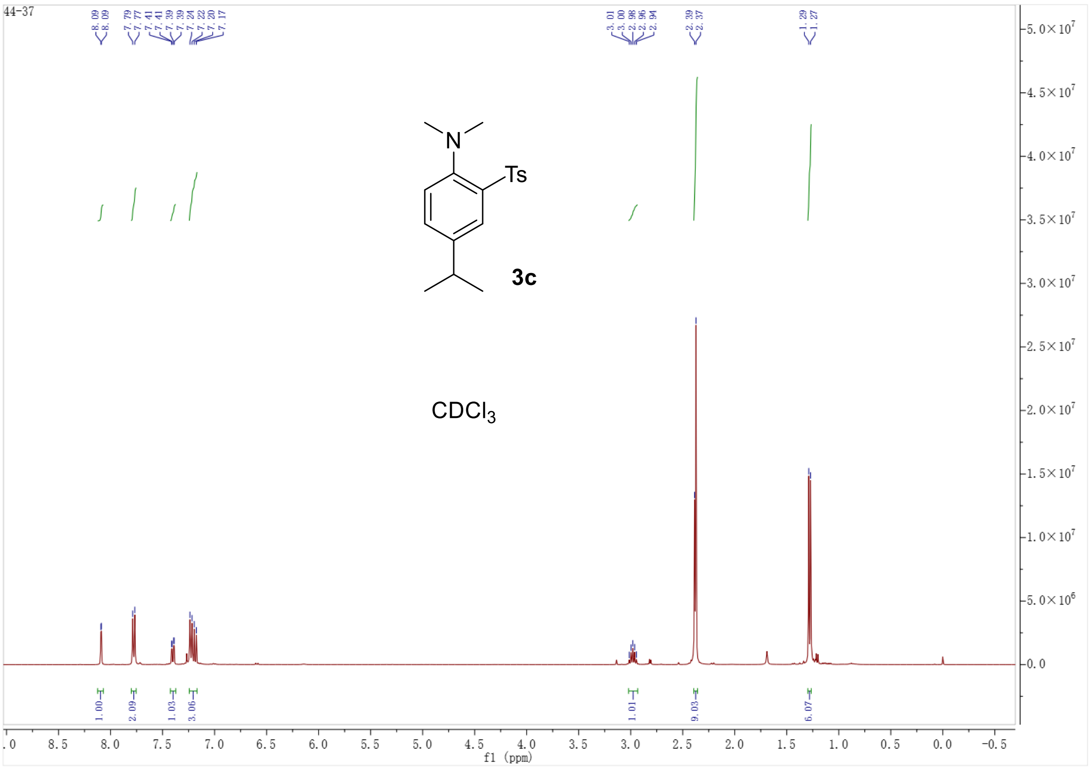
44-37
8.09 8.09 7.79 7.77 7.41 7.39 7.39 7.24 7.22 7.20 7.17
3.01 3.00 2.98 2.96 2.94 2.39 2.37 1.29 1.27
N
Ts
3c
CDCl3
1.00 2.09 1.03 3.06 1.01 9.03 6.07
f1 (ppm)

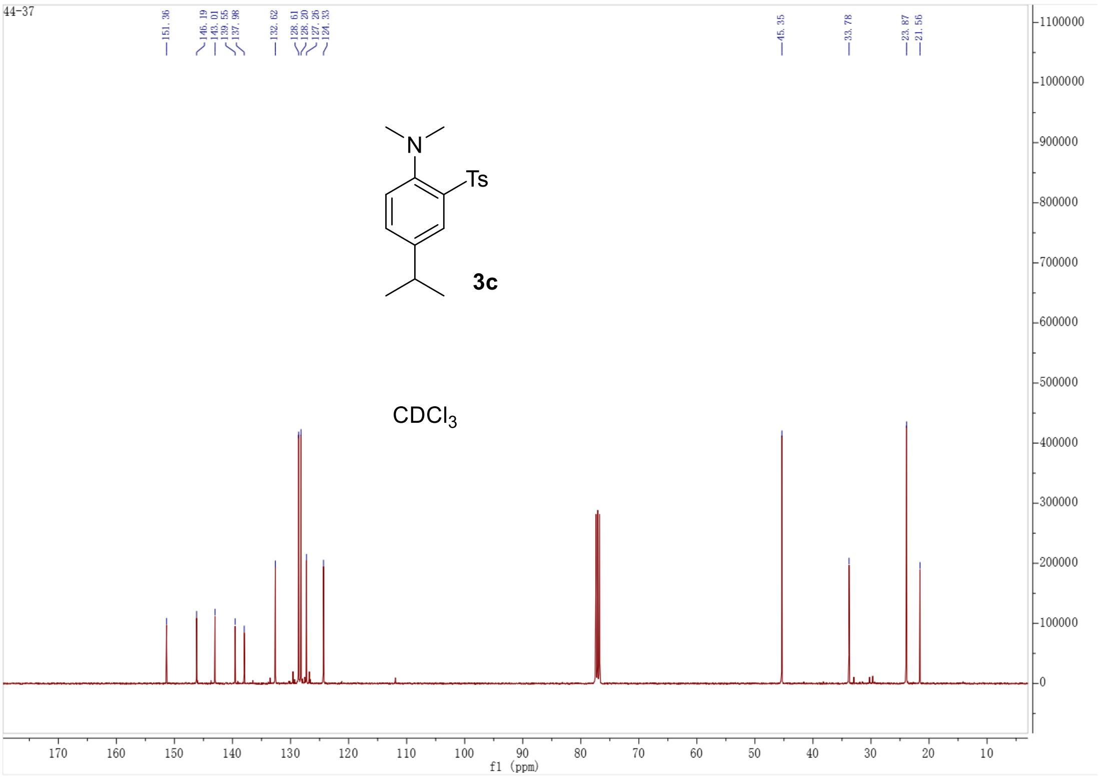
44-37
151.36 146.19 143.01 139.55 137.98 132.62 128.61 128.20 127.26 124.33
45.35 33.78 23.87 21.56
N
Ts
3c
CDCl3
f1 (ppm)

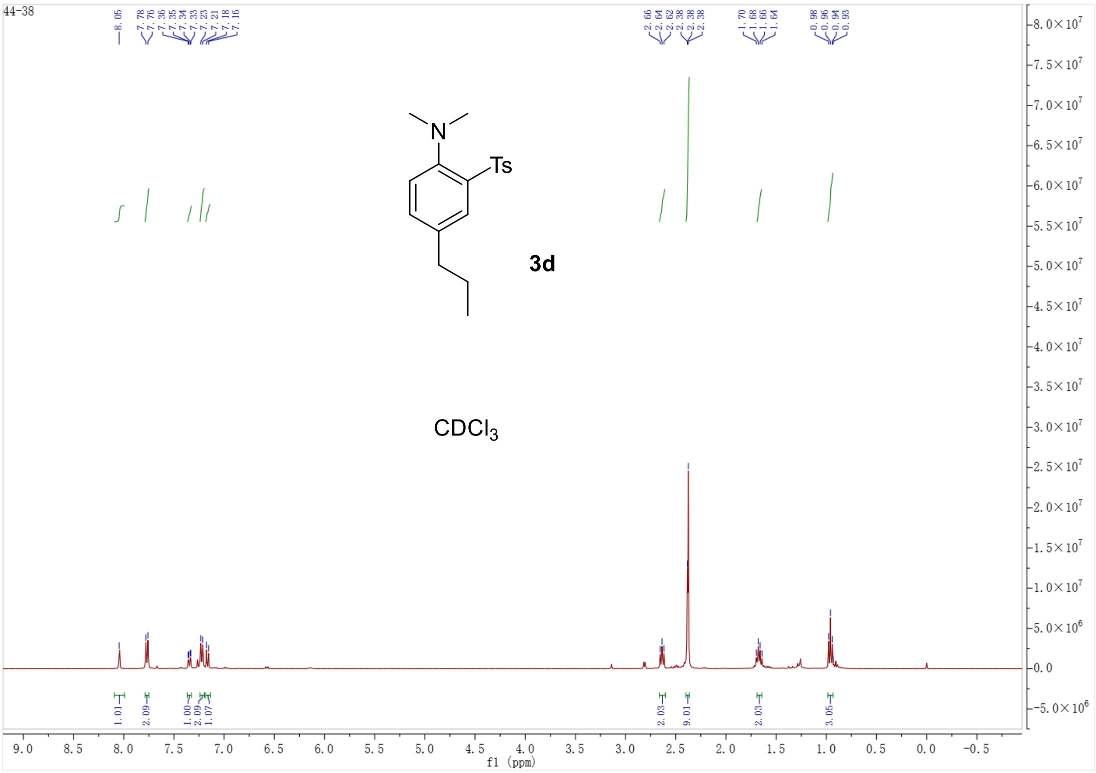
44-38
8.05
7.78
7.76
7.36
7.35
7.34
7.33
7.23
7.21
7.18
7.16
2.66
2.64
2.62
2.38
2.38
2.38
1.70
1.68
1.66
1.64
0.98
0.96
0.94
0.93
N
Ts
3d
CDCl3
1.01
2.09
1.00
2.09
1.07
2.03
9.01
2.03
3.05
f1 (ppm)

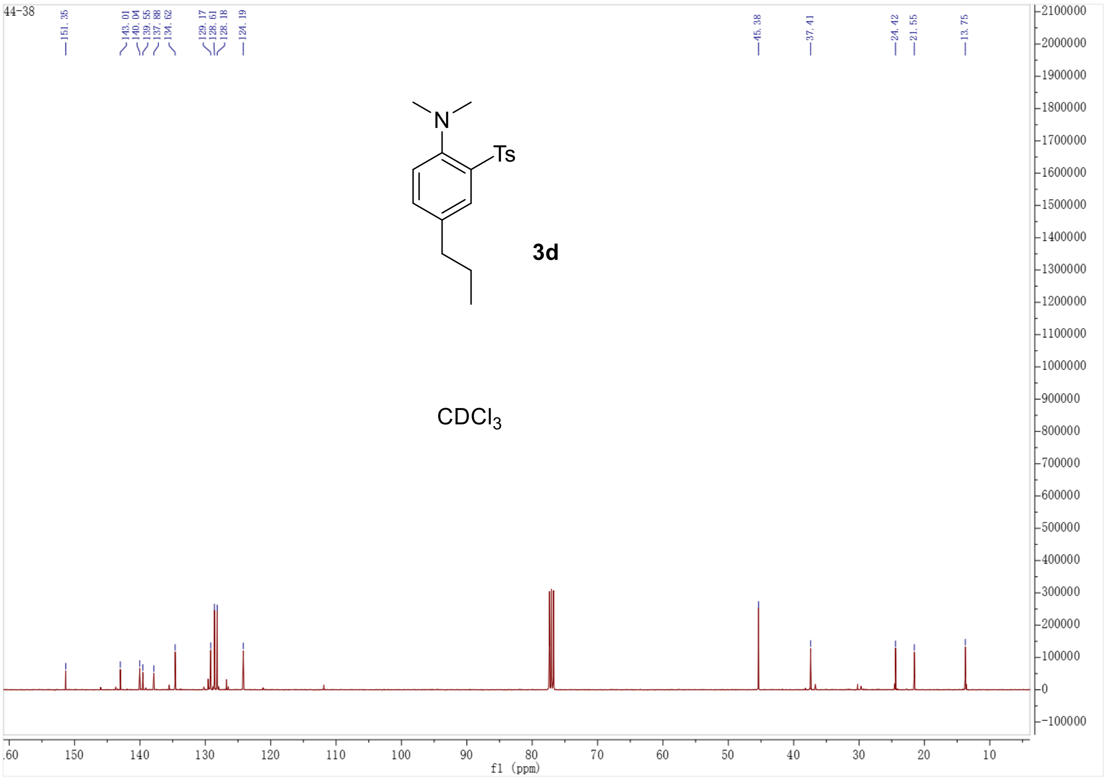
44-38
151.35
143.01
140.04
139.55
137.88
134.62
129.17
128.61
128.18
124.19
45.38
37.41
24.42
21.55
13.75
N
Ts
3d
CDCl3
f1 (ppm)

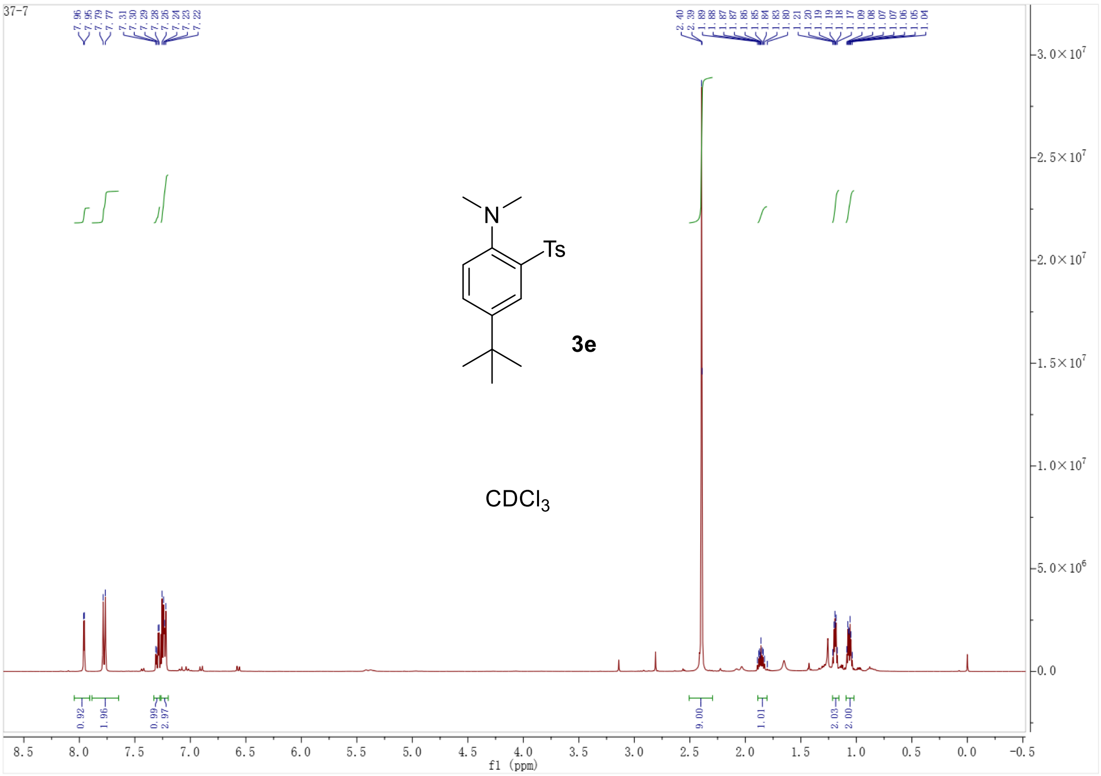
37-7
7.96 7.95 7.79 7.77 7.31 7.30 7.29 7.28 7.26 7.24 7.23 7.22
2.40 2.39 1.89 1.88 1.87 1.87 1.86 1.85 1.84 1.83 1.80 1.21 1.20 1.19 1.18 1.17 1.09 1.08 1.07 1.07 1.06 1.05 1.04
N
Ts
3e
CDCl$_3$
0.92 1.96 0.99 2.97 9.00 1.01 2.03 2.00
f1 (ppm)

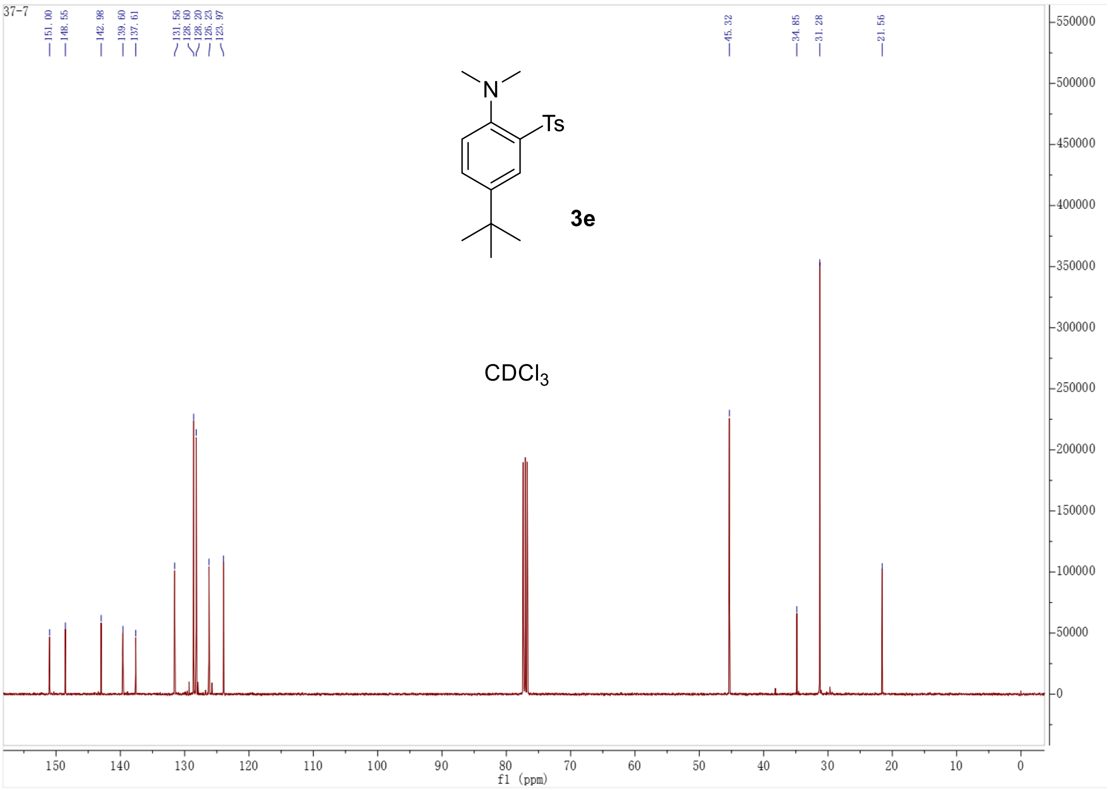
37-7
151.00 148.55 142.98 139.60 137.61 131.56 128.60 128.20 126.23 123.97
45.32 34.85 31.28 21.56
N
Ts
3e
CDCl$_3$
f1 (ppm)

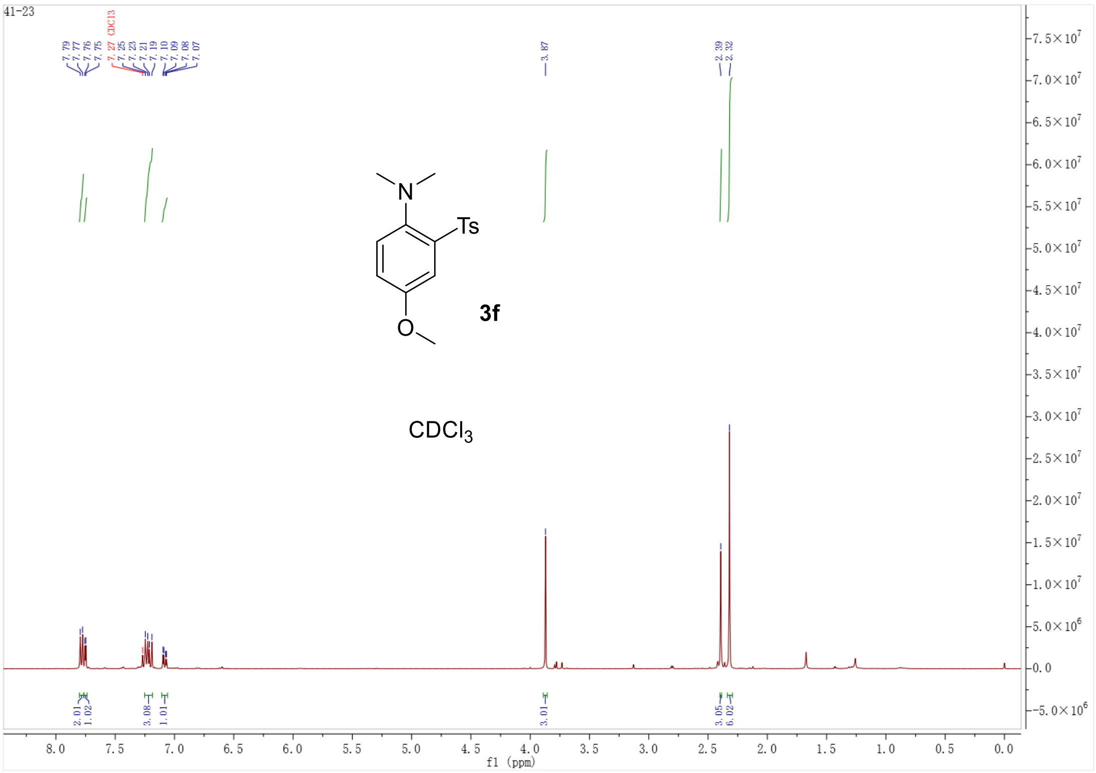
41-23
7.79
7.77
7.76
7.75
7.27 CDCl3
7.25
7.23
7.21
7.19
7.10
7.09
7.08
7.07
3.87
2.39
2.32
3f
CDCl3
2.01
1.02
3.08
1.01
3.01
3.05
6.02
f1 (ppm)

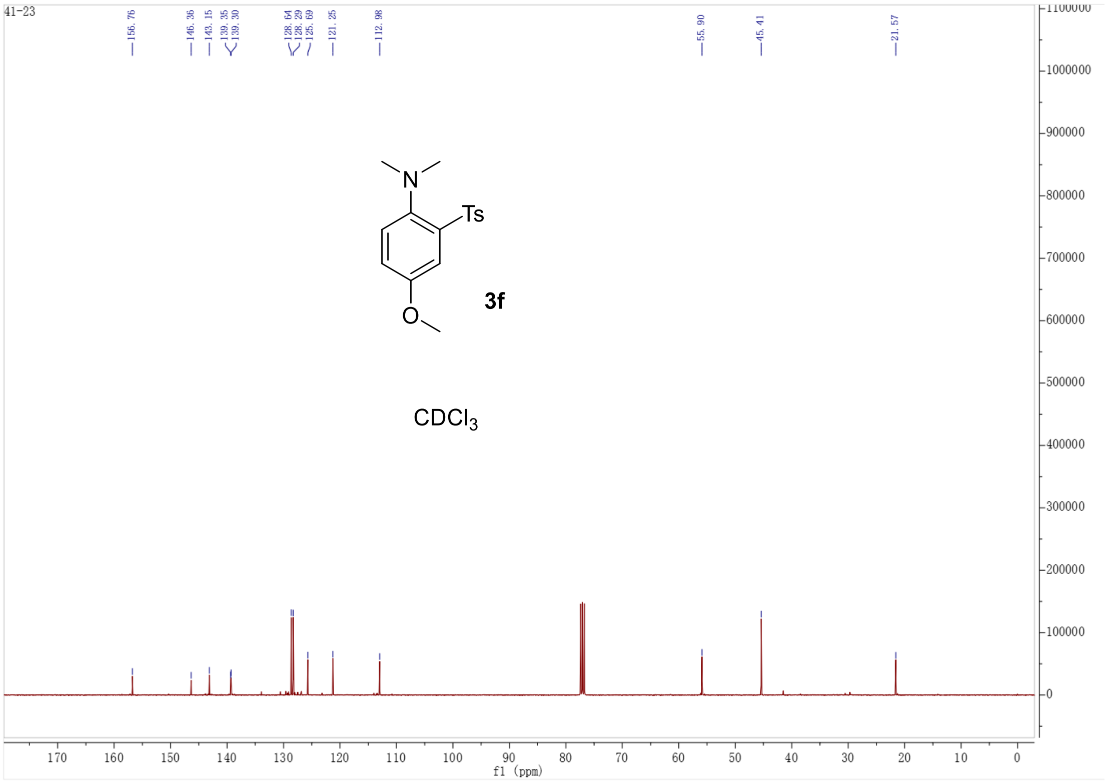
41-23
156.76
146.36
143.15
139.35
139.30
128.64
128.29
125.69
121.25
112.98
55.90
45.41
21.57
3f
CDCl3
f1 (ppm)

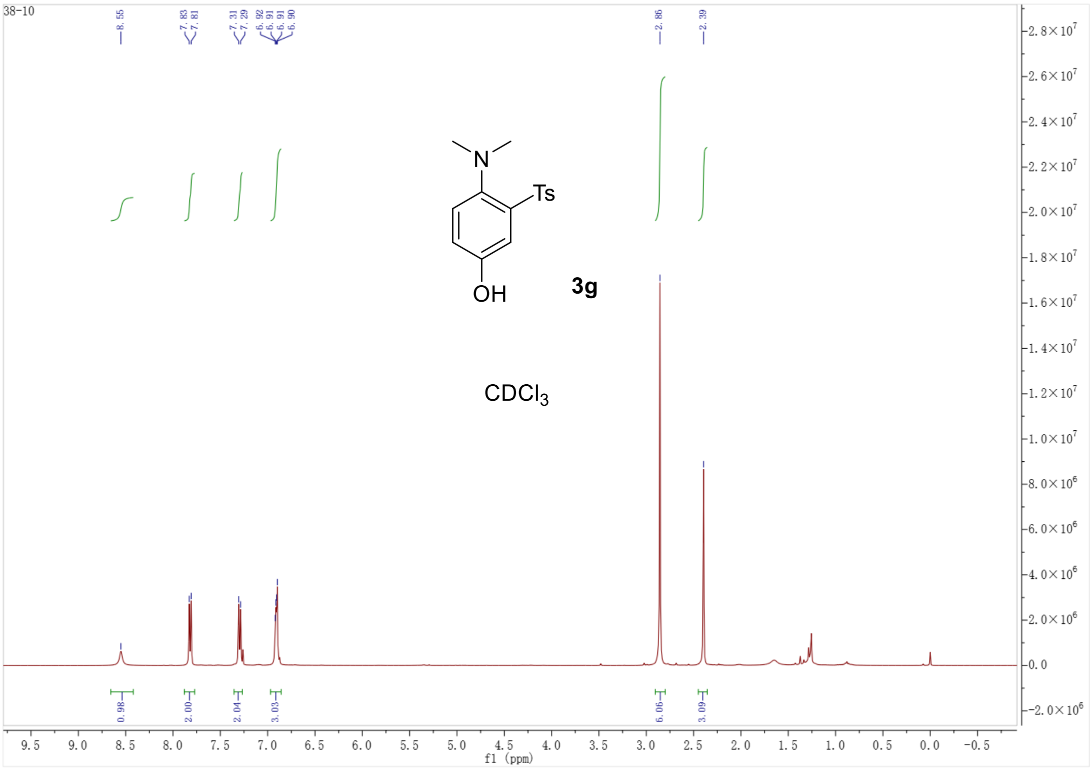
38-10
8.55
7.83
7.81
7.31
7.29
6.92
6.91
6.91
6.90
2.86
2.39
Ts
N
OH
3g
CDCl3
0.98
2.00
2.04
3.03
6.06
3.09
f1 (ppm)

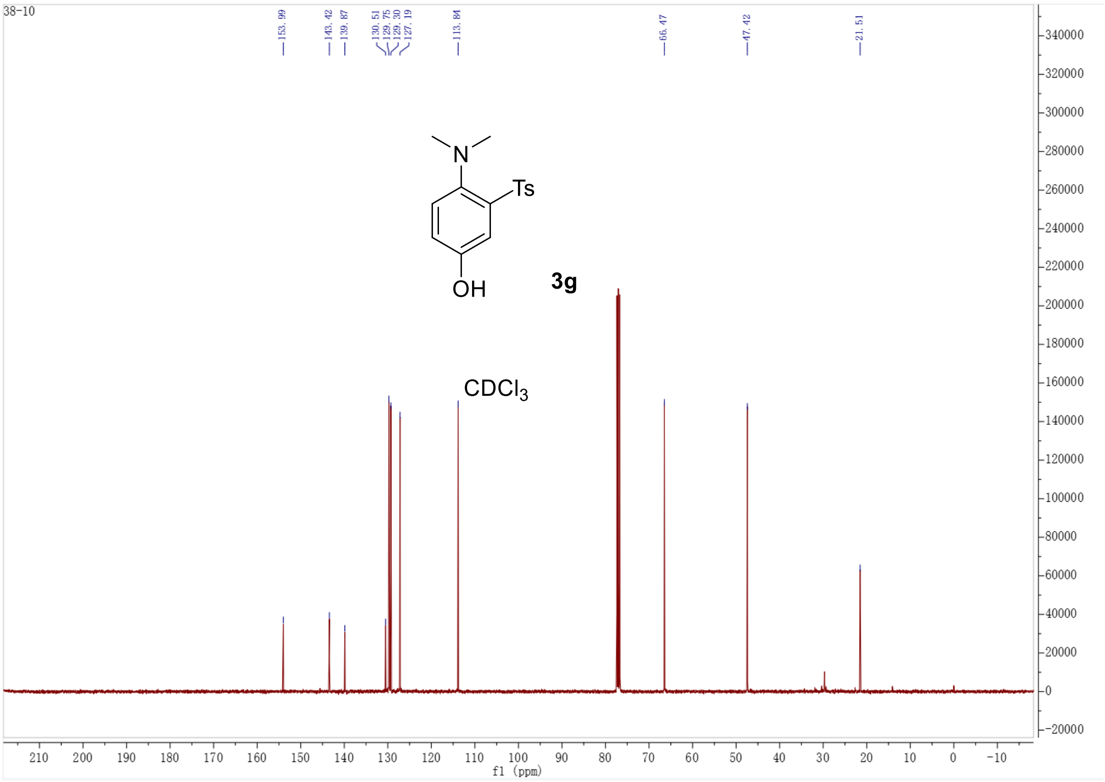
38-10
153.99
143.42
139.87
130.51
129.75
129.30
127.19
113.84
66.47
47.42
21.51
Ts
N
OH
3g
CDCl3
f1 (ppm)

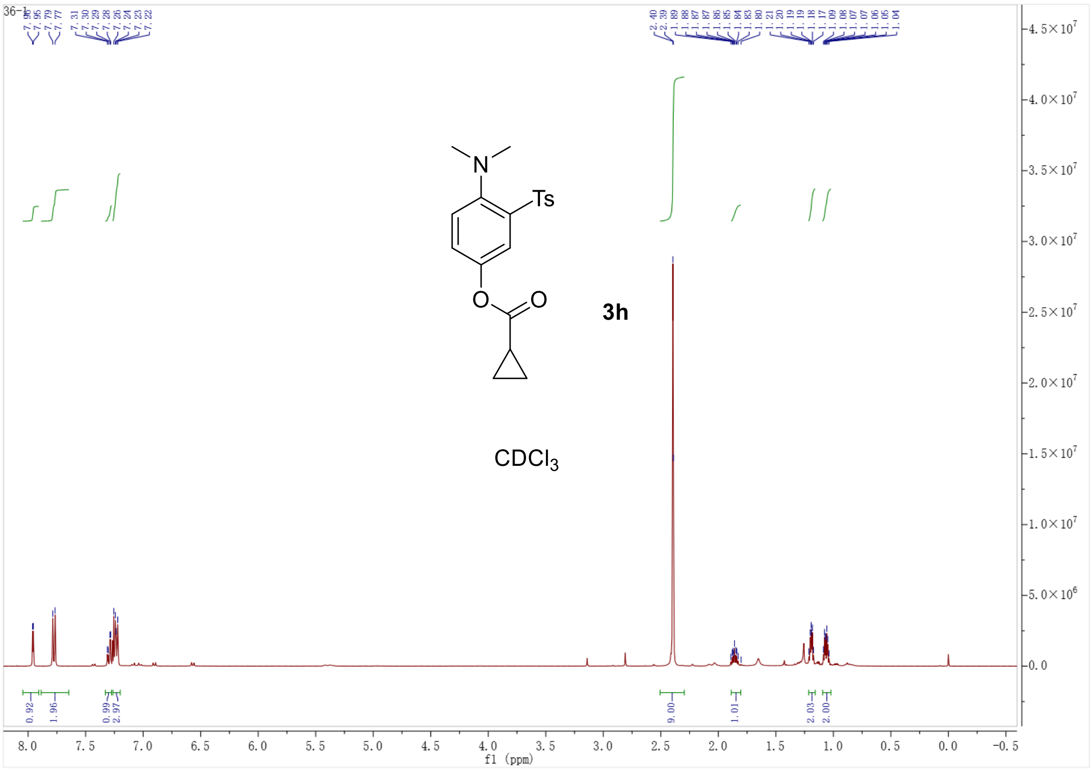
36-1
3h
CDCl3
f1 (ppm)

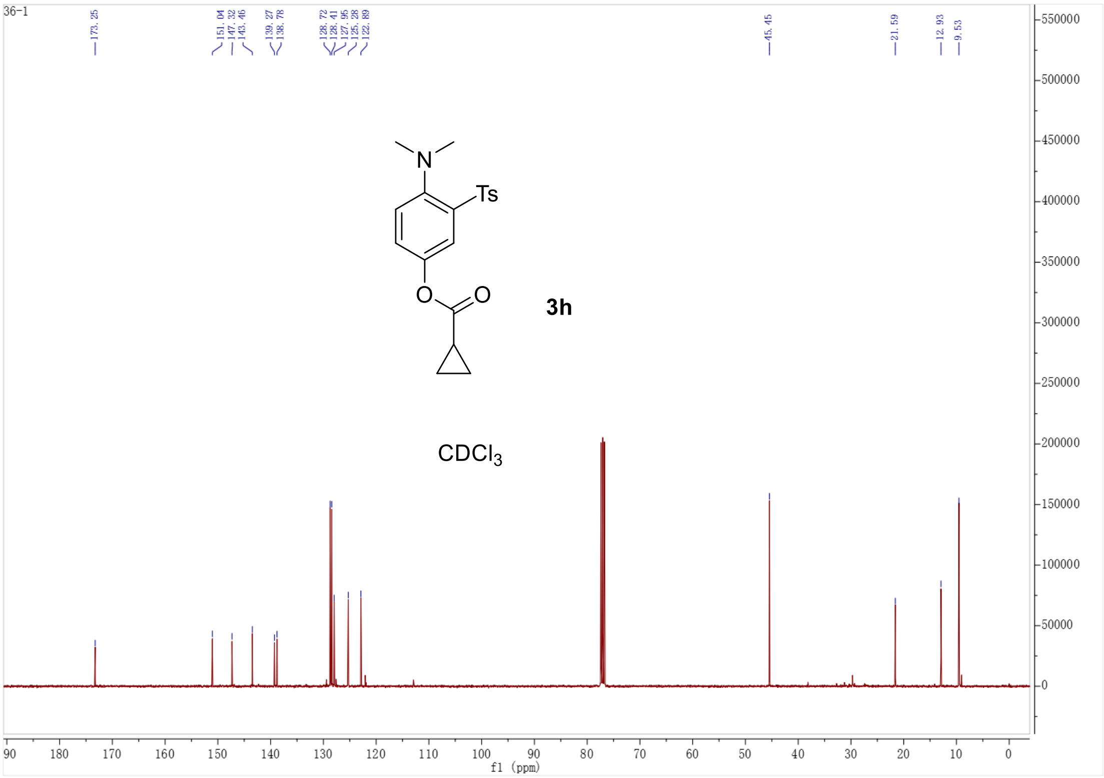
36-1
3h
CDCl3
f1 (ppm)

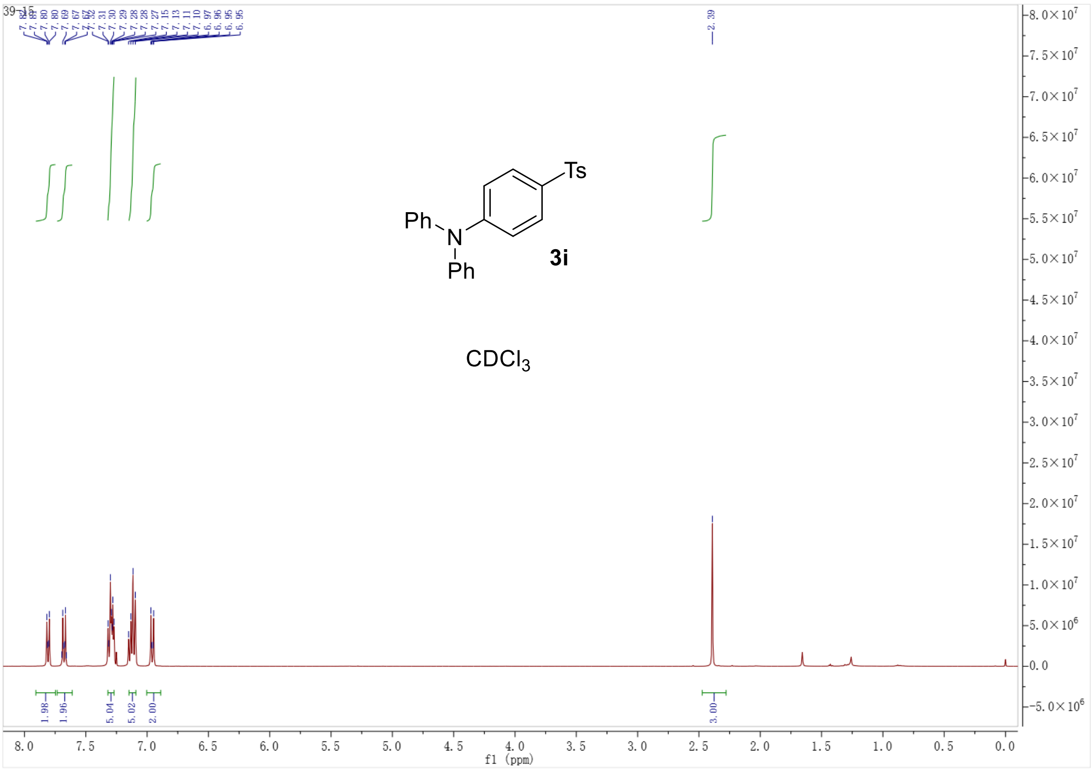
39-15
2.39
Ph
N
Ph
Ts
3i
CDCl3
1.98
1.96
5.04
5.02
2.00
3.00
f1 (ppm)

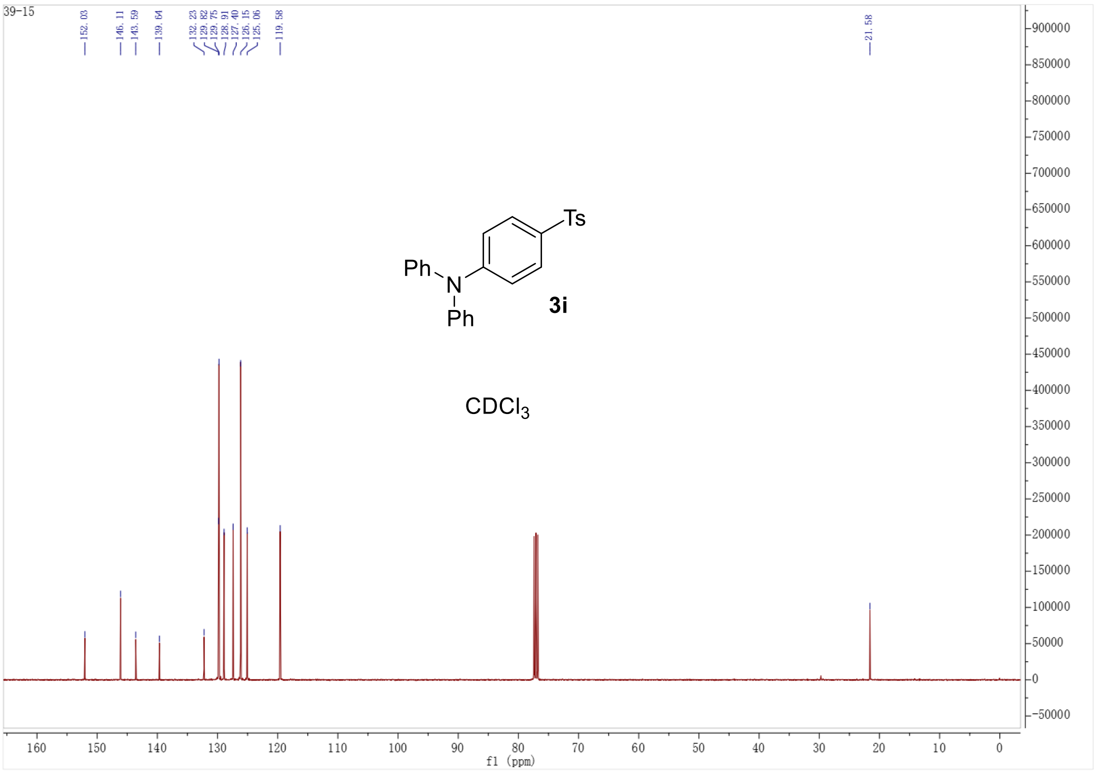
39-15
152.03
146.11
143.59
139.64
132.23
129.82
129.75
128.91
127.40
126.15
125.06
119.58
21.58
Ph
N
Ph
Ts
3i
CDCl3
f1 (ppm)

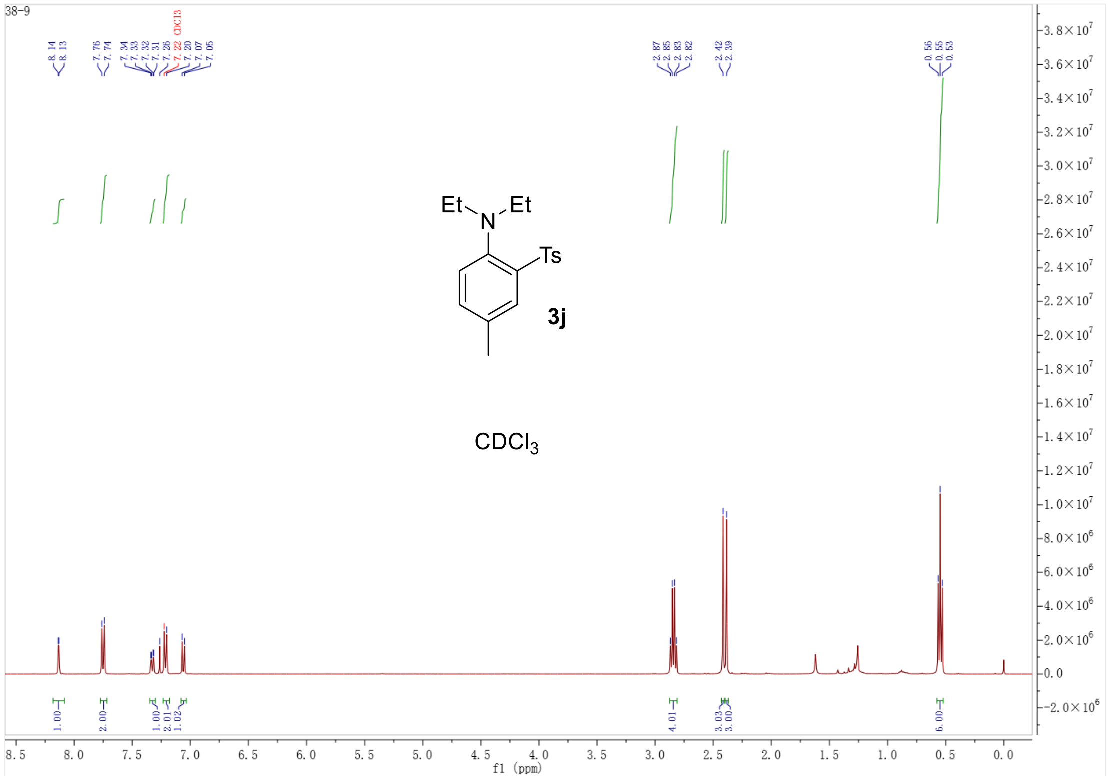
38-9
8.14
8.13
7.76
7.74
7.34
7.33
7.32
7.31
7.26
7.22 CDCl3
7.20
7.07
7.05
2.87
2.85
2.83
2.82
2.42
2.39
0.56
0.55
0.53
Et
N
Et
Ts
3j
CDCl3
1.00
2.00
1.00
2.01
1.02
4.01
3.03
3.00
6.00
f1 (ppm)

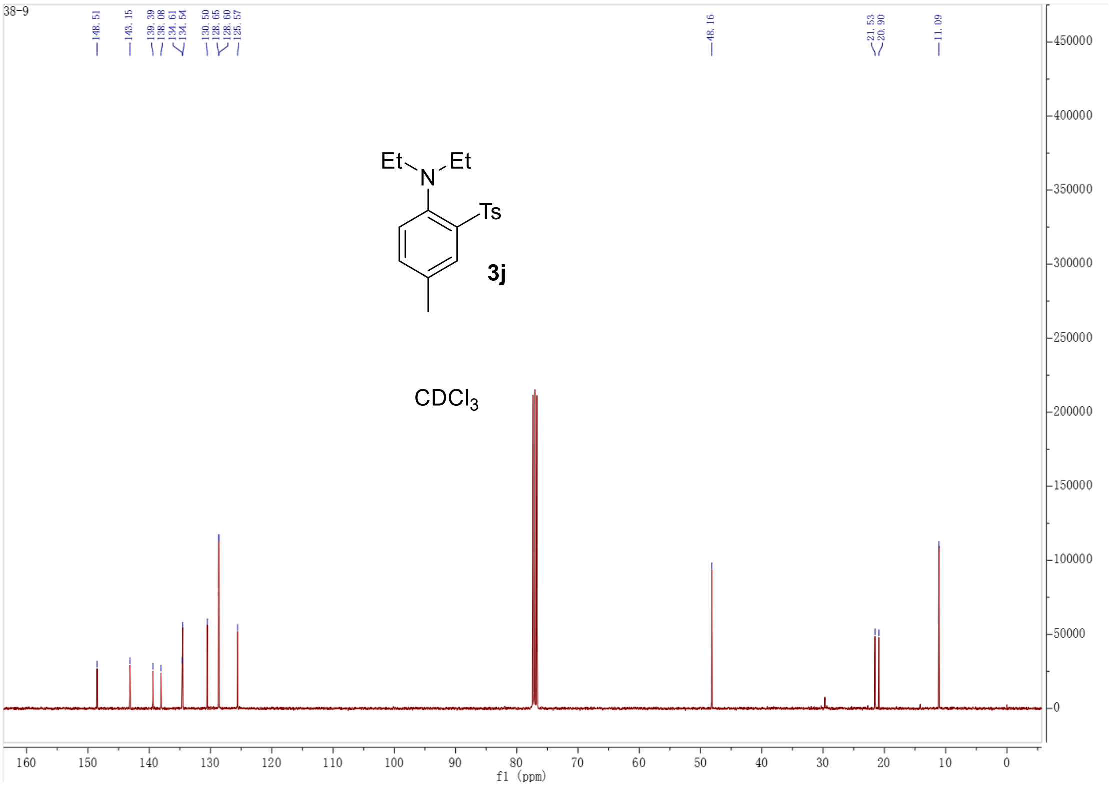
38-9
148.51
143.15
139.39
138.08
134.61
134.54
130.50
128.65
128.60
125.57
48.16
21.53
20.90
11.09
Et
N
Et
Ts
3j
CDCl3
f1 (ppm)

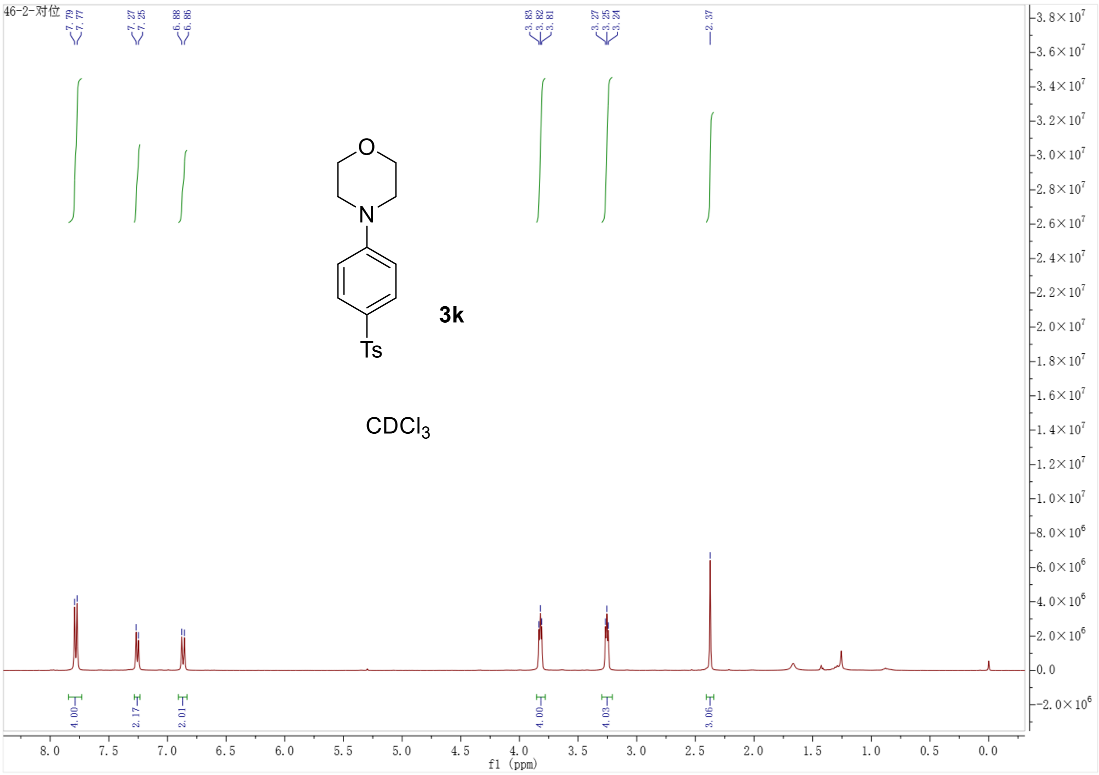
46-2-对位
7.79
7.77
7.27
7.25
6.88
6.86
3.83
3.82
3.81
3.27
3.25
3.24
2.37
O
N
Ts
3k
CDCl3
4.00
2.17
2.01
4.00
4.03
3.06
f1 (ppm)

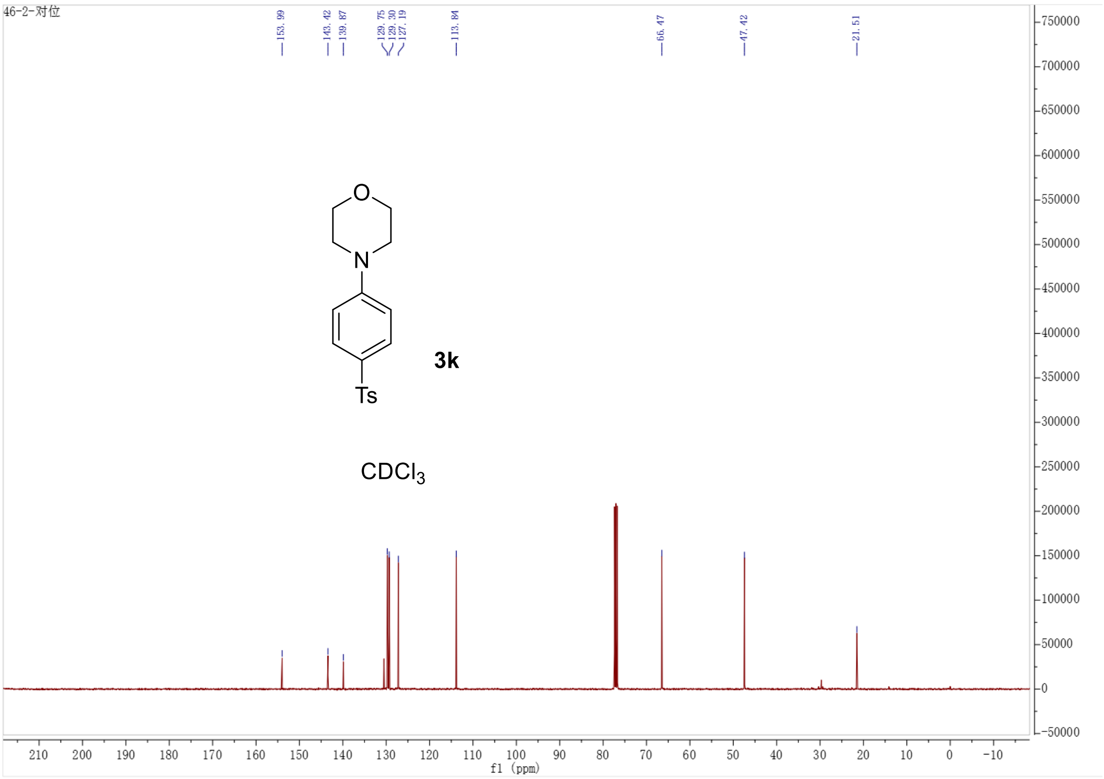
46-2-对位
153.99
143.42
139.87
129.75
129.30
127.19
113.84
66.47
47.42
21.51
O
N
Ts
3k
CDCl3
f1 (ppm)

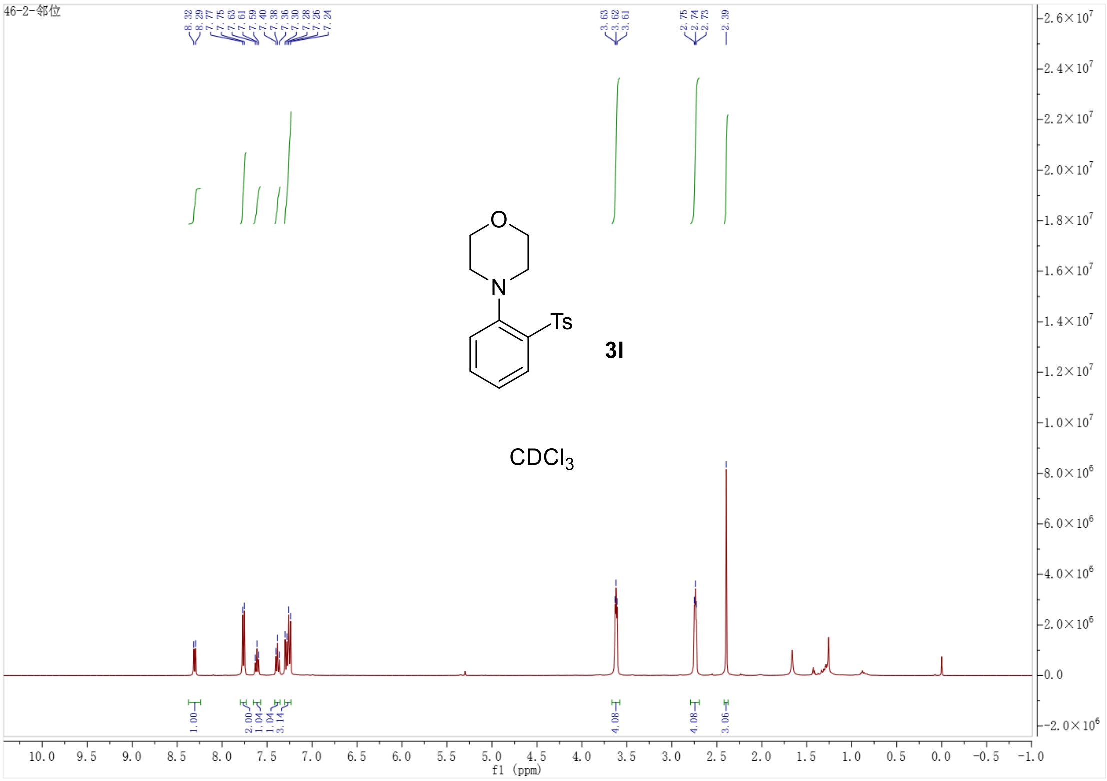
46-2-邻位
3l
CDCl3
f1 (ppm)

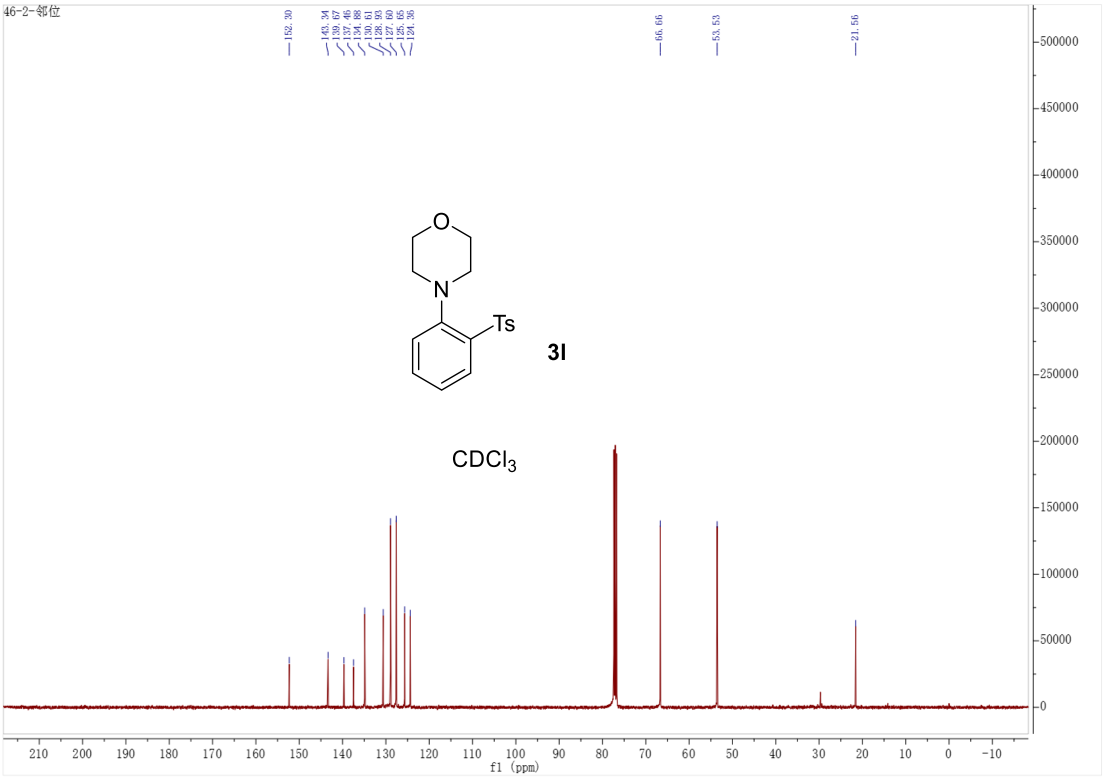
46-2-邻位
3l
CDCl3
f1 (ppm)

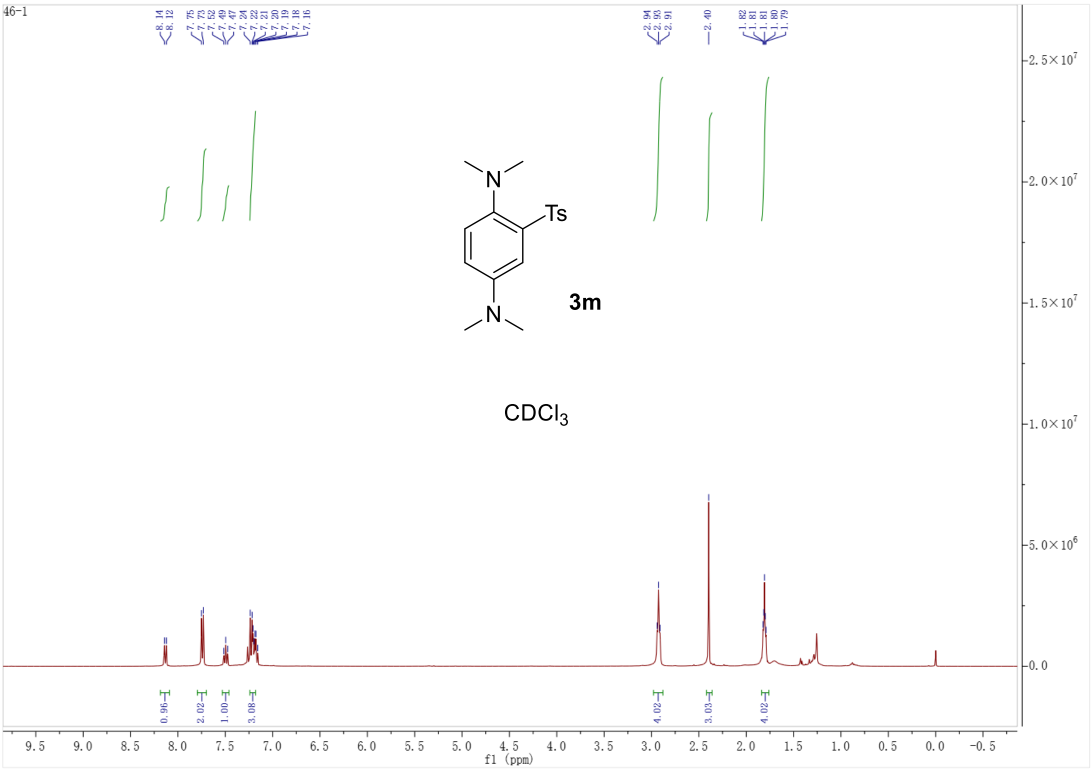
46-1
8.14
8.12
7.75
7.73
7.52
7.49
7.47
7.24
7.22
7.21
7.20
7.19
7.18
7.16
2.94
2.93
2.91
2.40
1.82
1.81
1.81
1.80
1.79
N
Ts
N
3m
CDCl3
0.96
2.02
1.00
3.08
4.02
3.03
4.02
f1 (ppm)

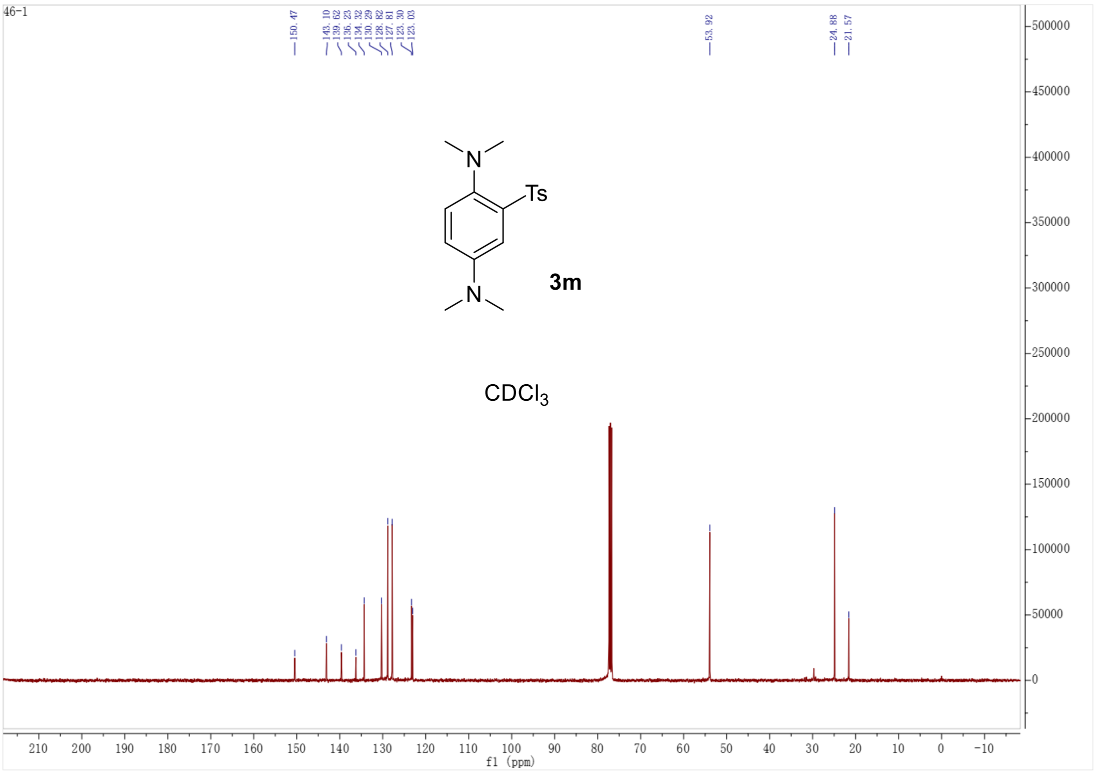
46-1
150.47
143.10
139.62
136.23
134.32
130.29
128.82
127.81
123.30
123.03
53.92
24.88
21.57
N
Ts
N
3m
CDCl3
f1 (ppm)

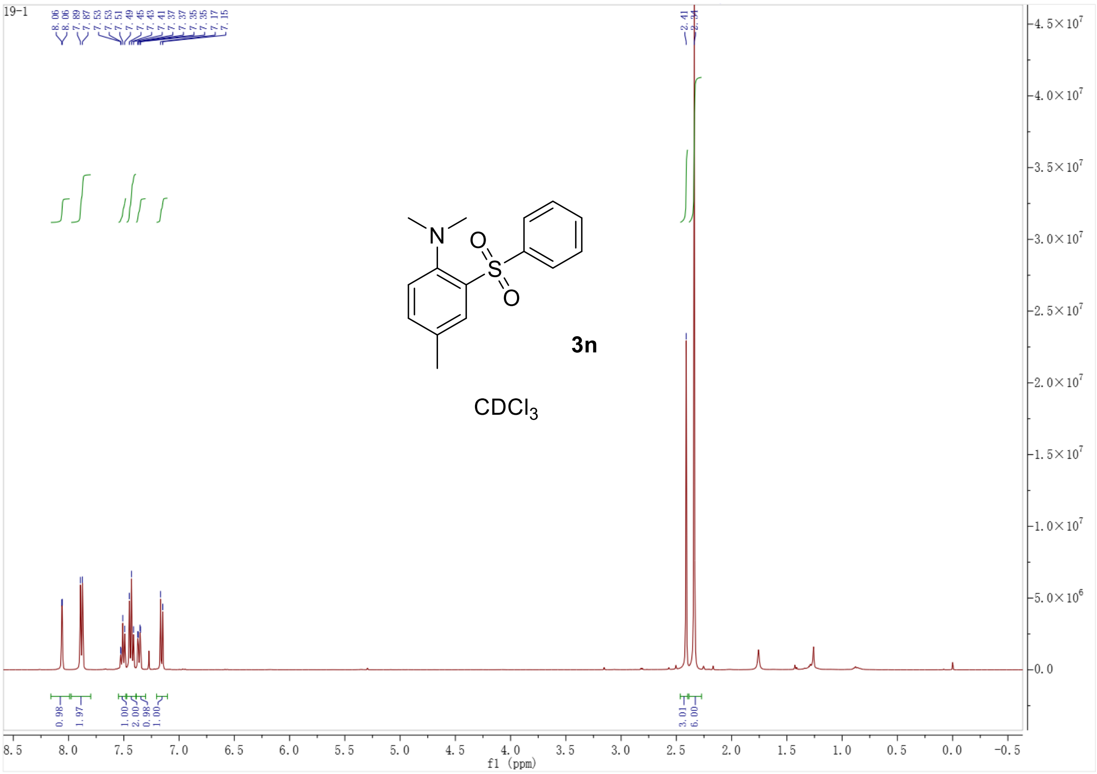
19-1
8.06
8.06
7.89
7.87
7.53
7.53
7.51
7.49
7.45
7.43
7.41
7.37
7.37
7.35
7.35
7.17
7.15
2.41
2.34
3n
CDCl3
0.98
1.97
1.00
2.00
0.98
1.00
3.01
6.00
f1 (ppm)

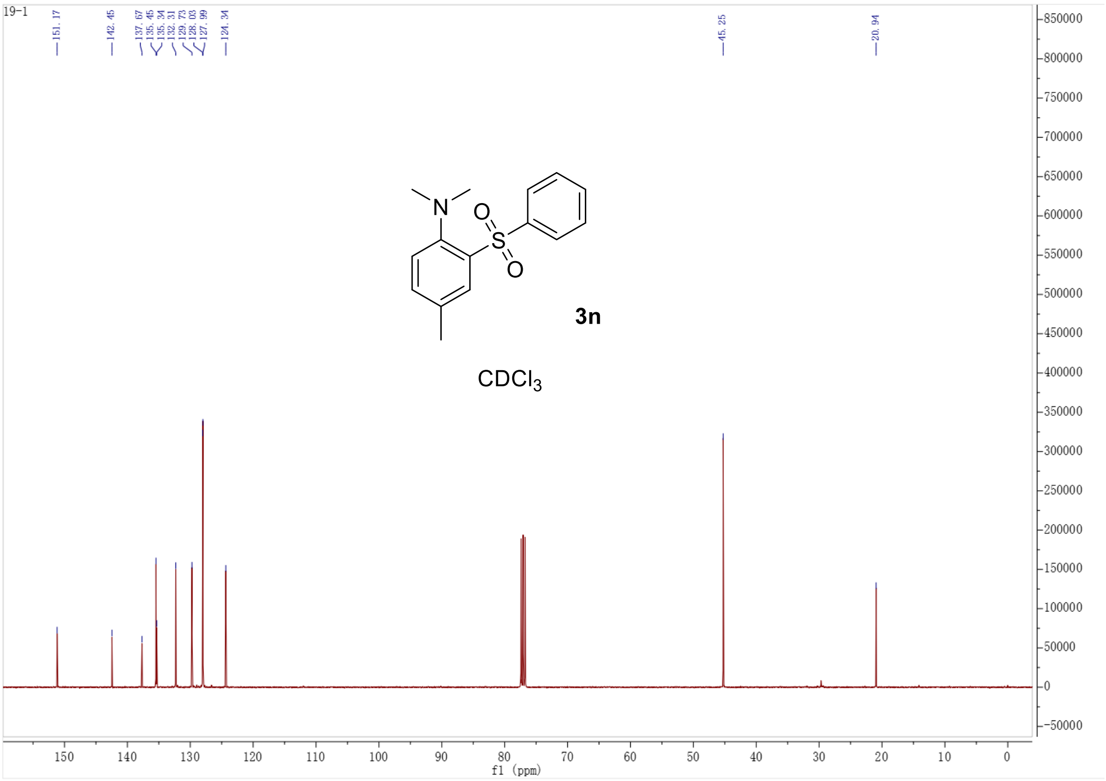
19-1
151.17
142.45
137.67
135.45
135.34
132.31
129.73
128.03
127.99
124.34
45.25
20.94
3n
CDCl3
f1 (ppm)

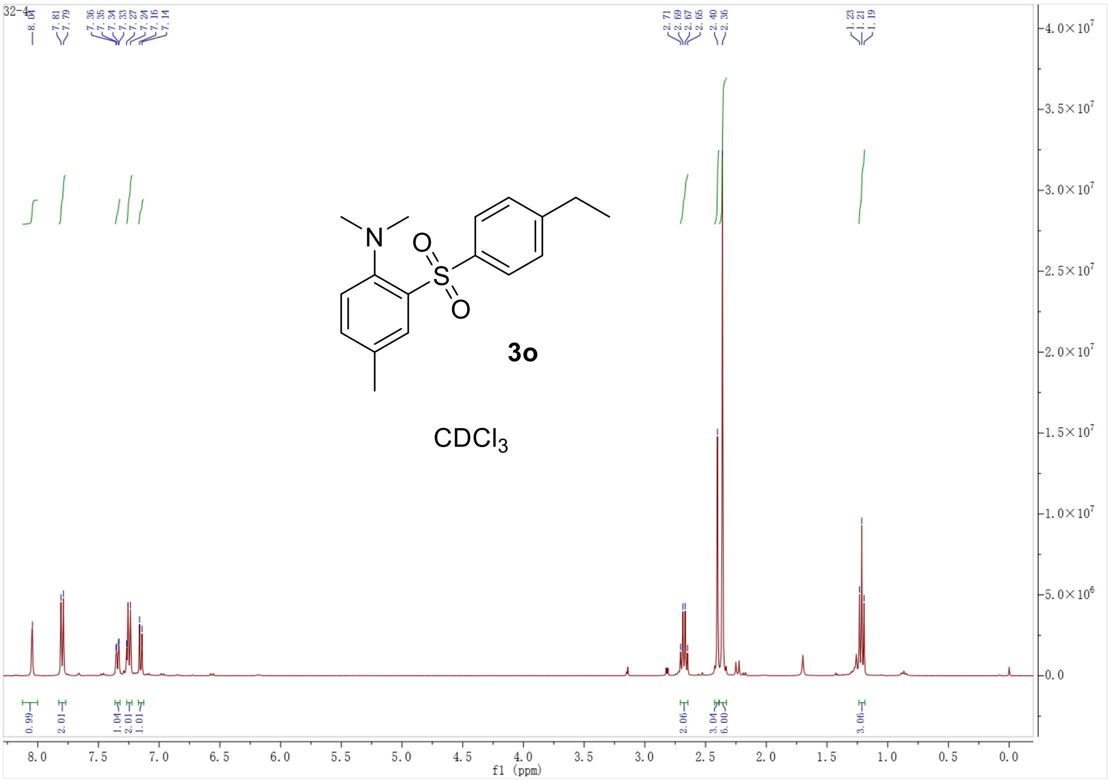

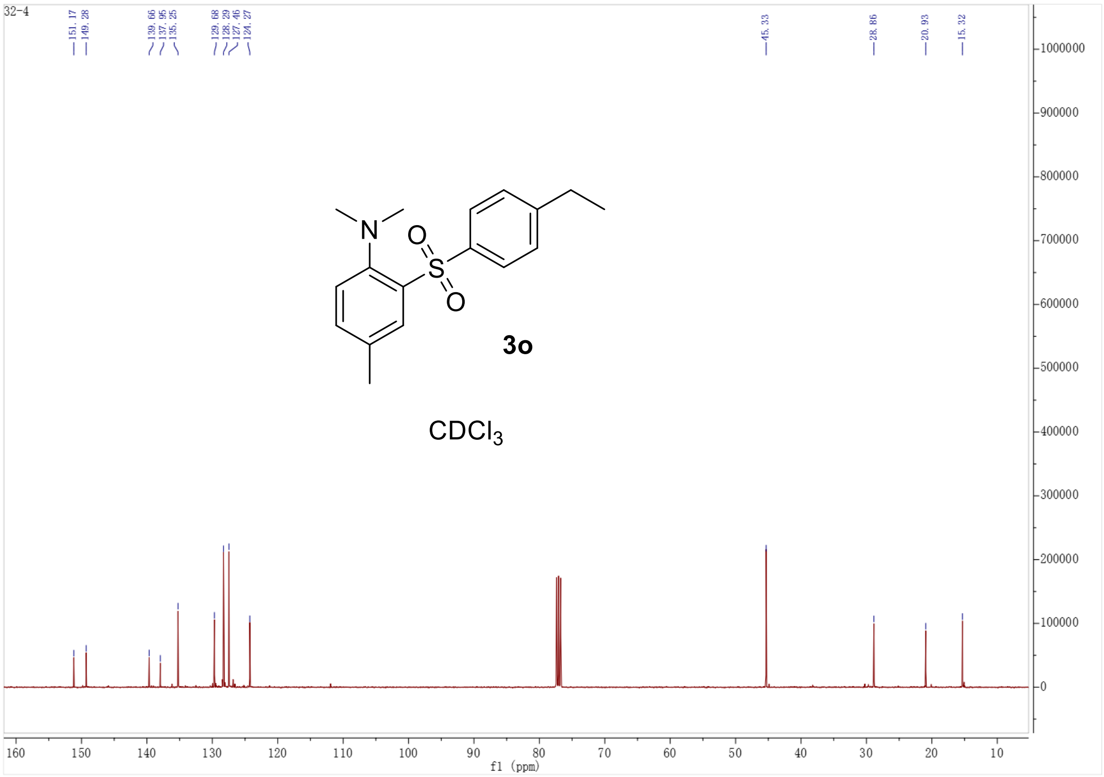

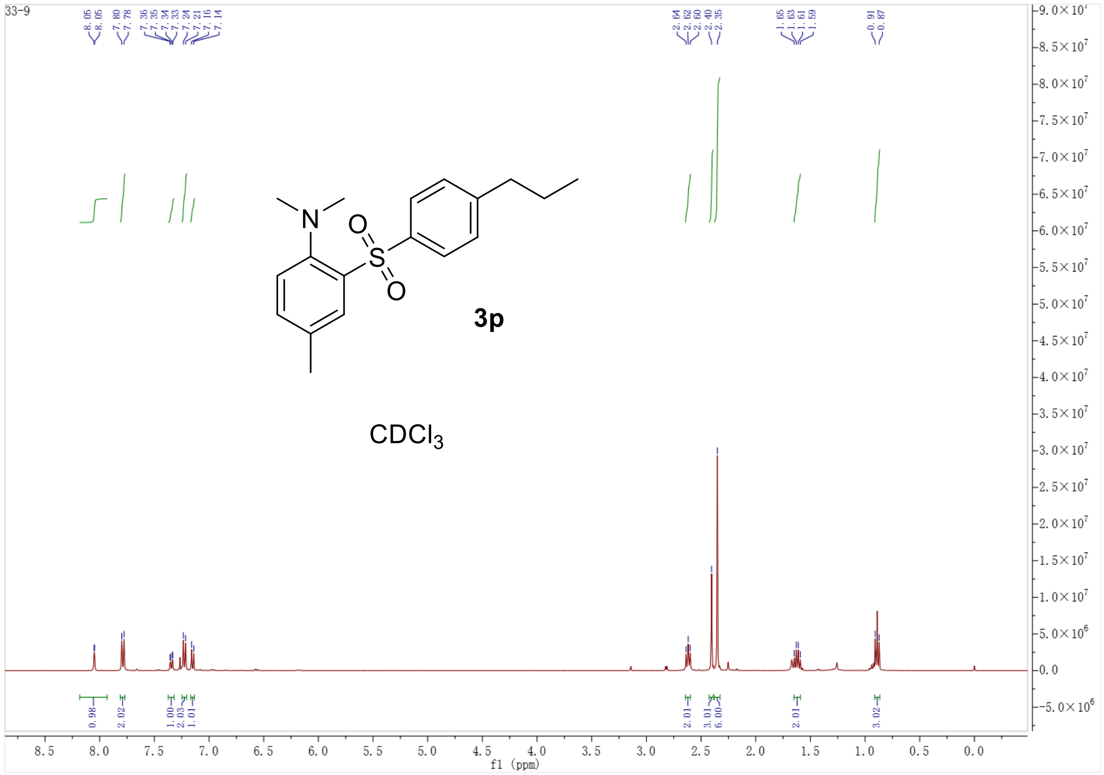
33-9
3p
CDCl3
f1 (ppm)

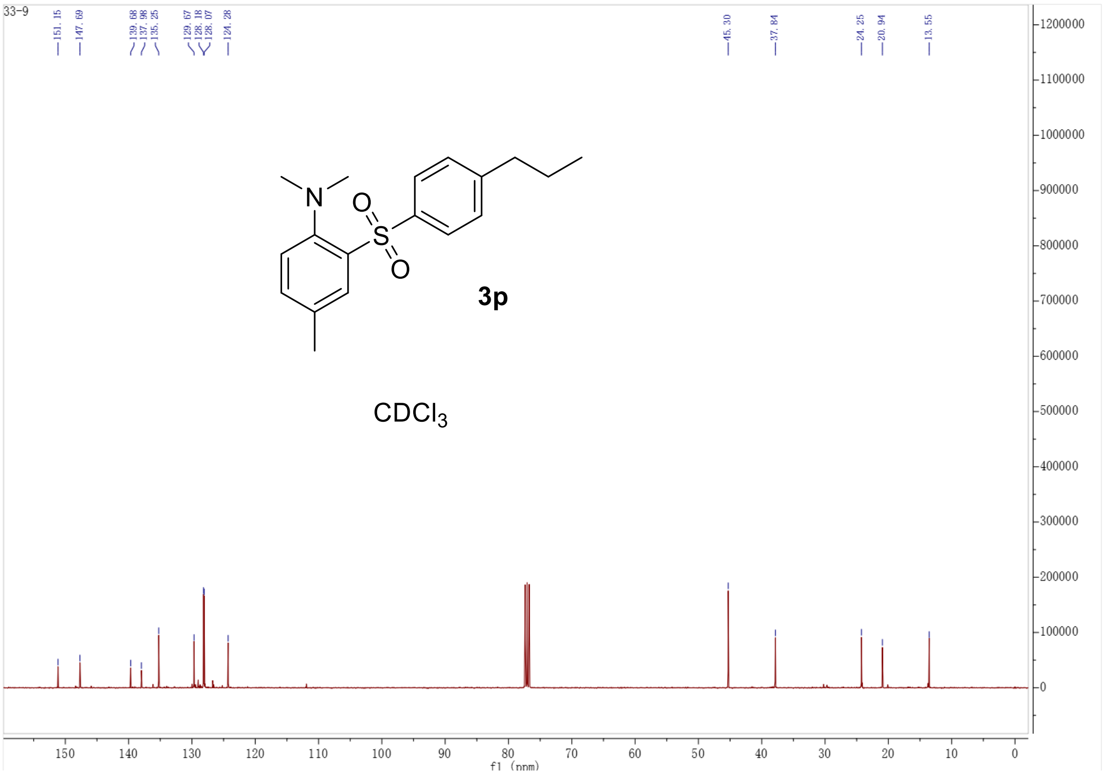
33-9
3p
CDCl3
f1 (ppm)

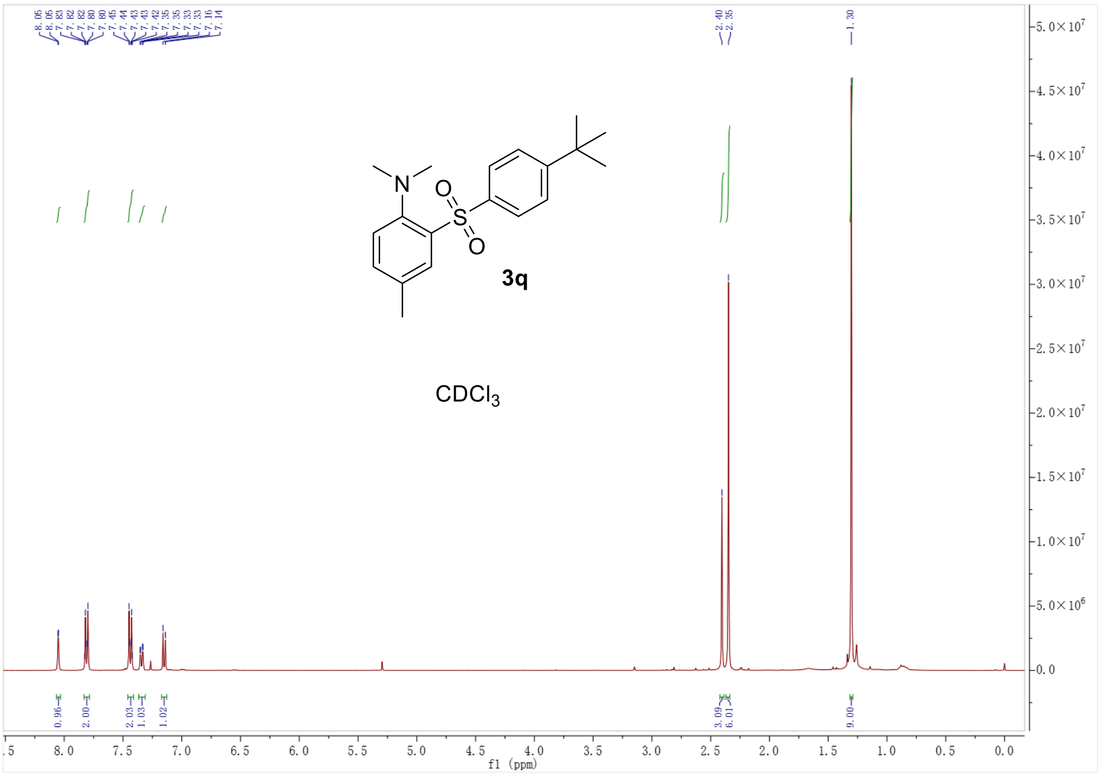
3q
CDCl3

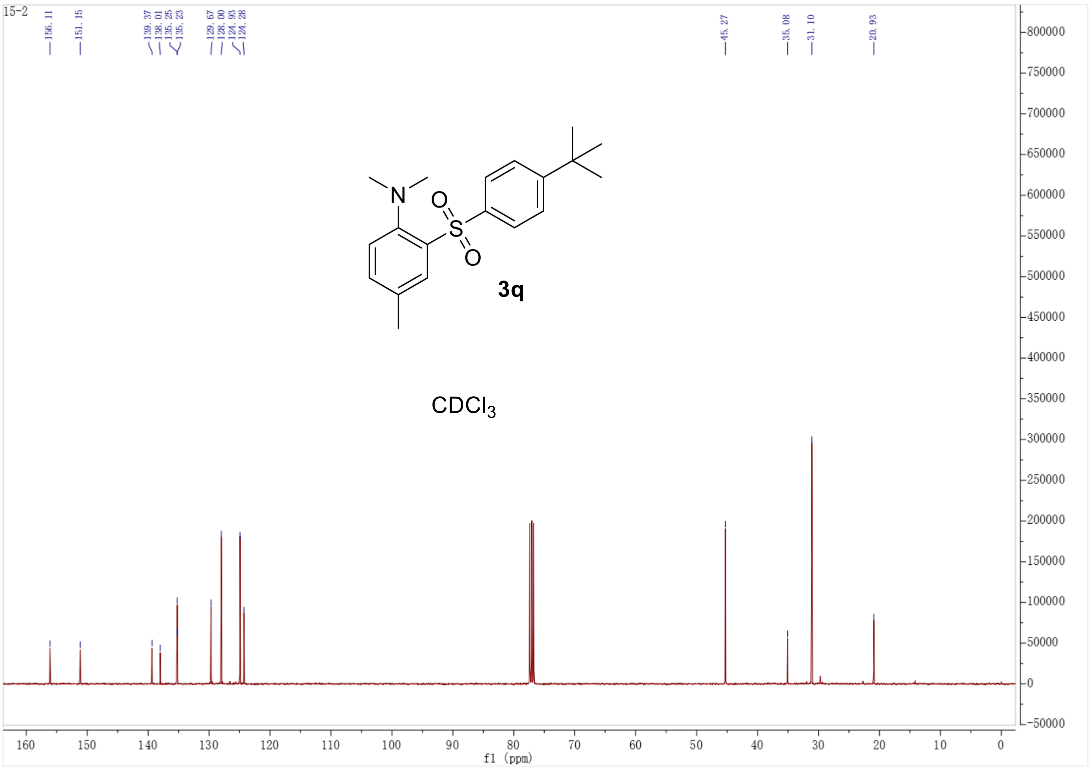
3q
CDCl3

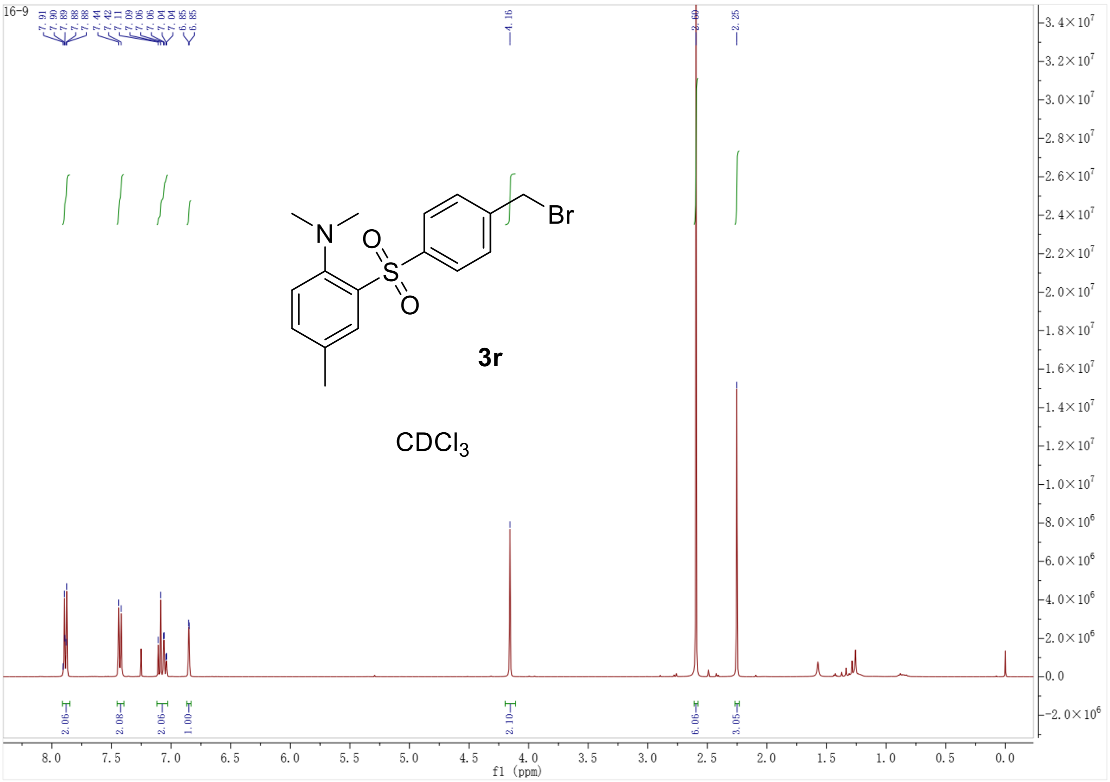
16-9
7.91
7.90
7.89
7.88
7.88
7.44
7.42
7.11
7.09
7.06
7.06
7.04
7.04
6.85
6.85
4.16
2.60
2.25
N
O
S
O
Br
3r
CDCl3
2.06
2.08
2.06
1.00
2.10
6.06
3.05
f1 (ppm)

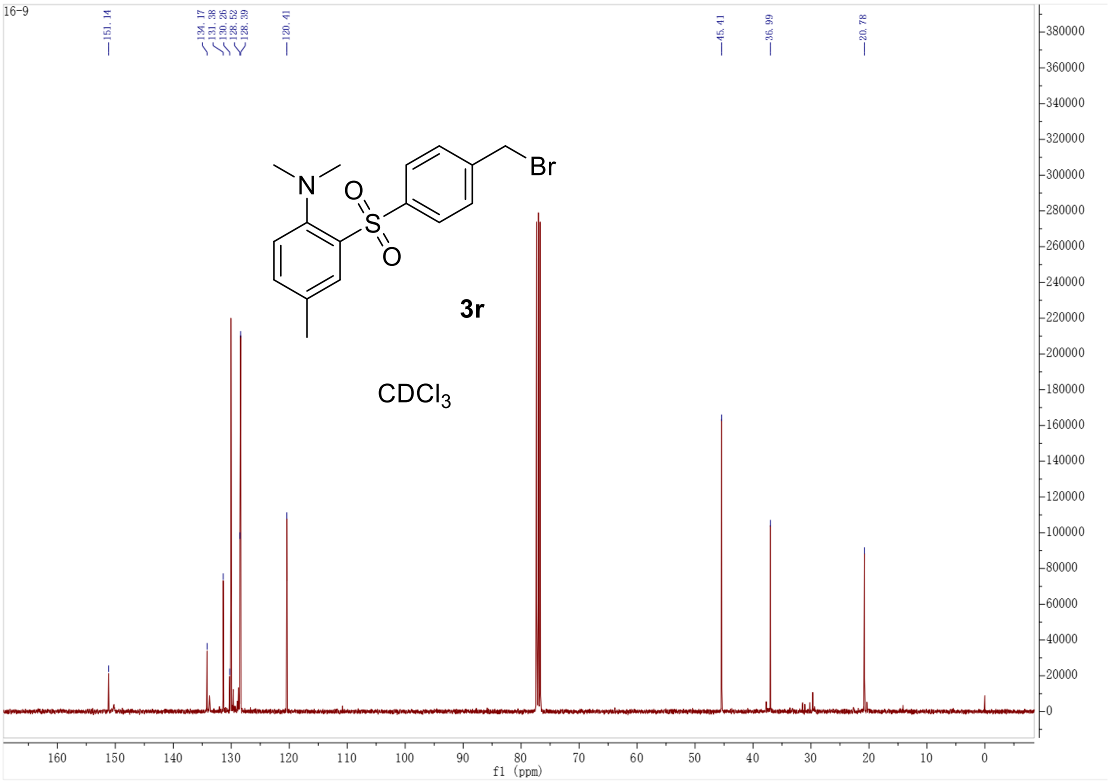
16-9
151.14
134.17
131.38
130.26
128.52
128.39
120.41
45.41
36.99
20.78
N
O
S
O
Br
3r
CDCl3
f1 (ppm)

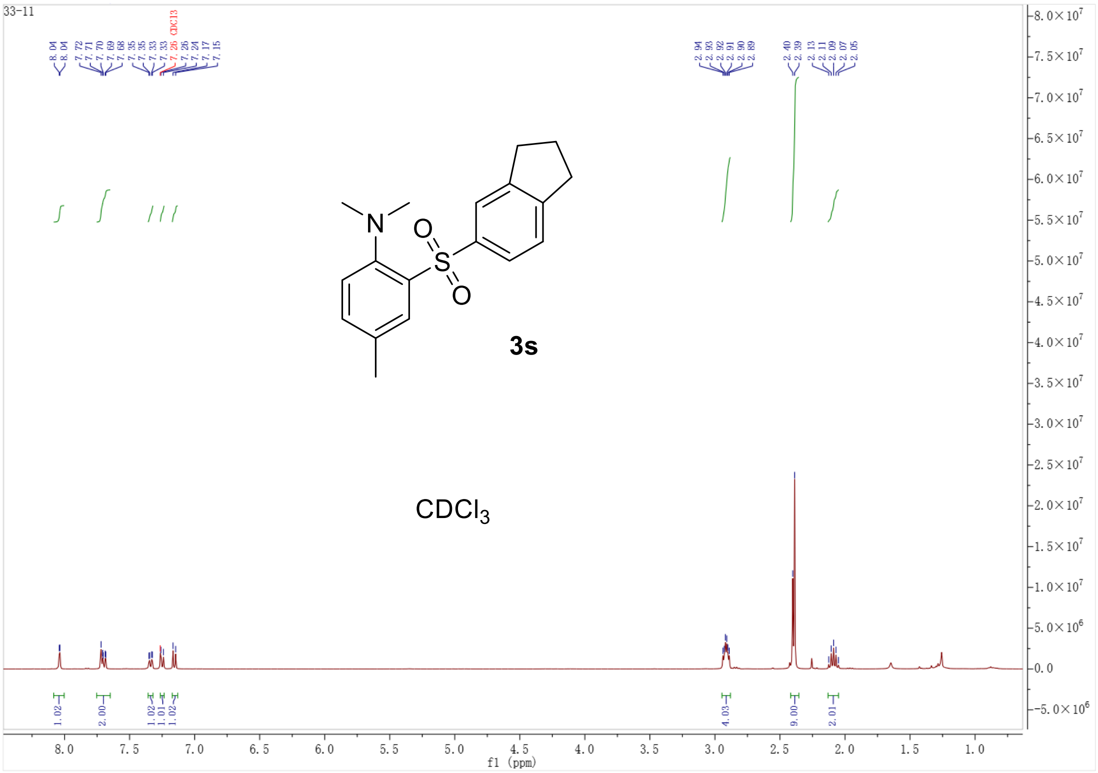
33-11
3s
CDCl3

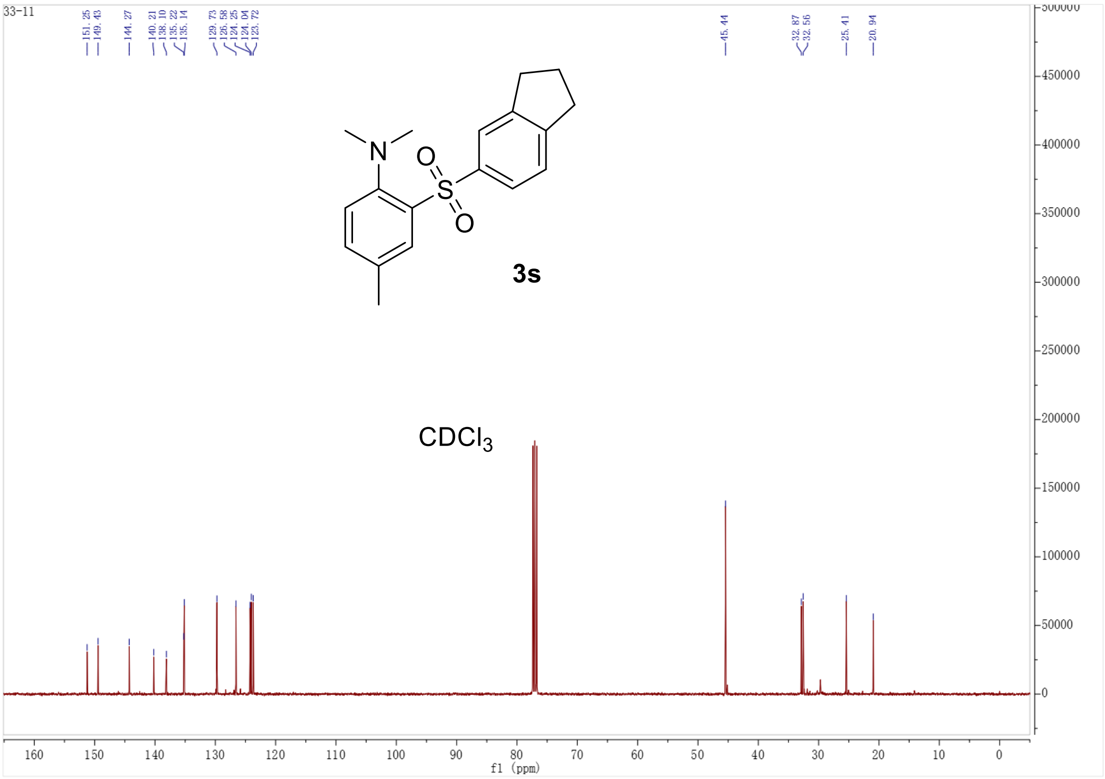
33-11
3s
CDCl3

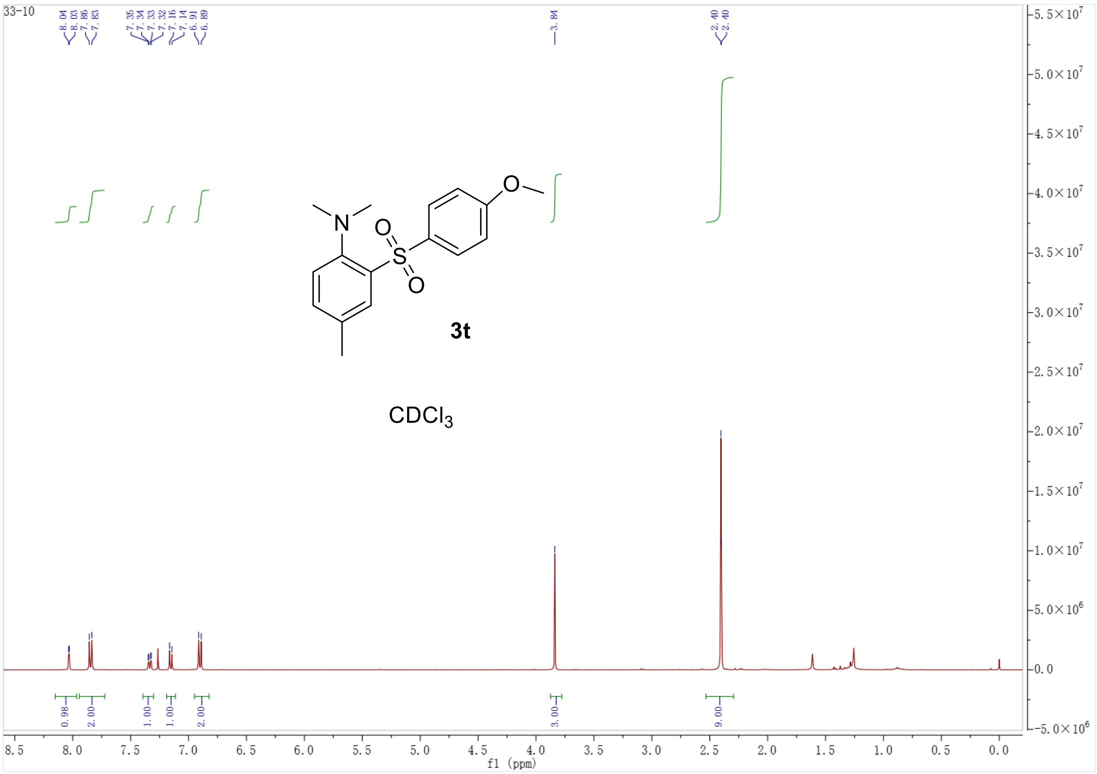
33-10
8.04
8.03
7.86
7.83
7.35
7.34
7.33
7.32
7.16
7.14
6.91
6.89
3.84
2.40
2.40
3t
CDCl3
0.98
2.00
1.00
1.00
2.00
3.00
9.00
f1 (ppm)

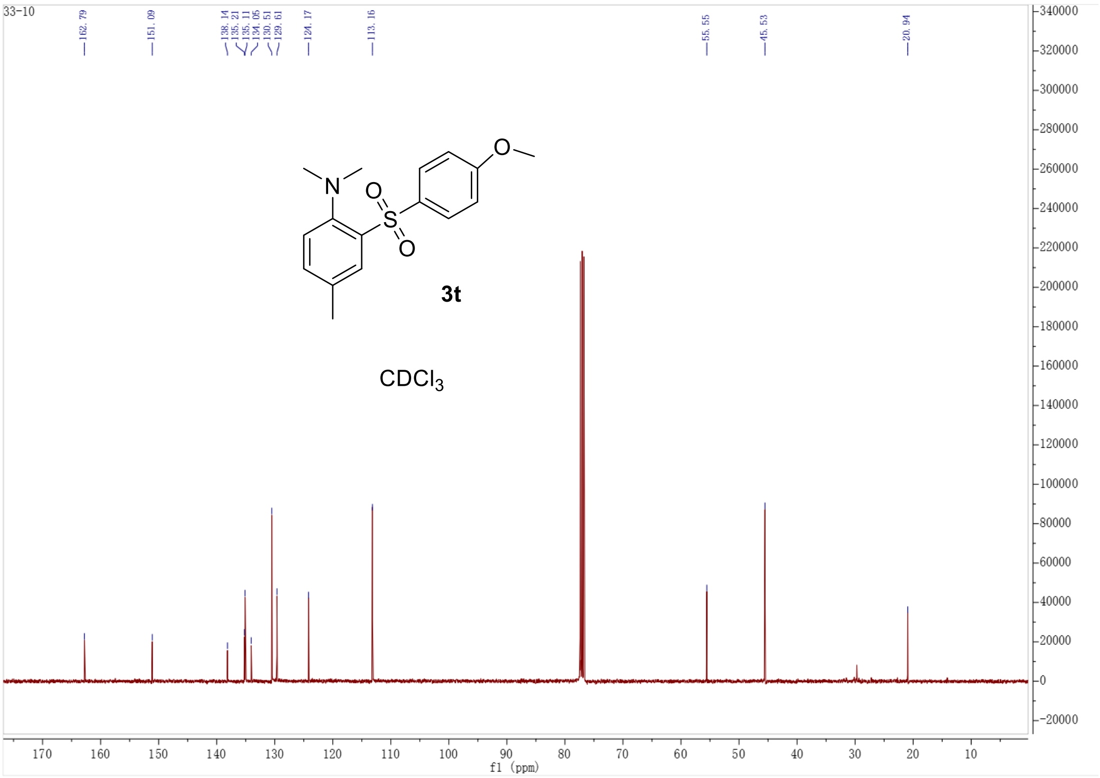
33-10
162.79
151.09
138.14
135.21
135.11
134.05
130.51
129.61
124.17
113.16
55.55
45.53
20.94
3t
CDCl3
f1 (ppm)

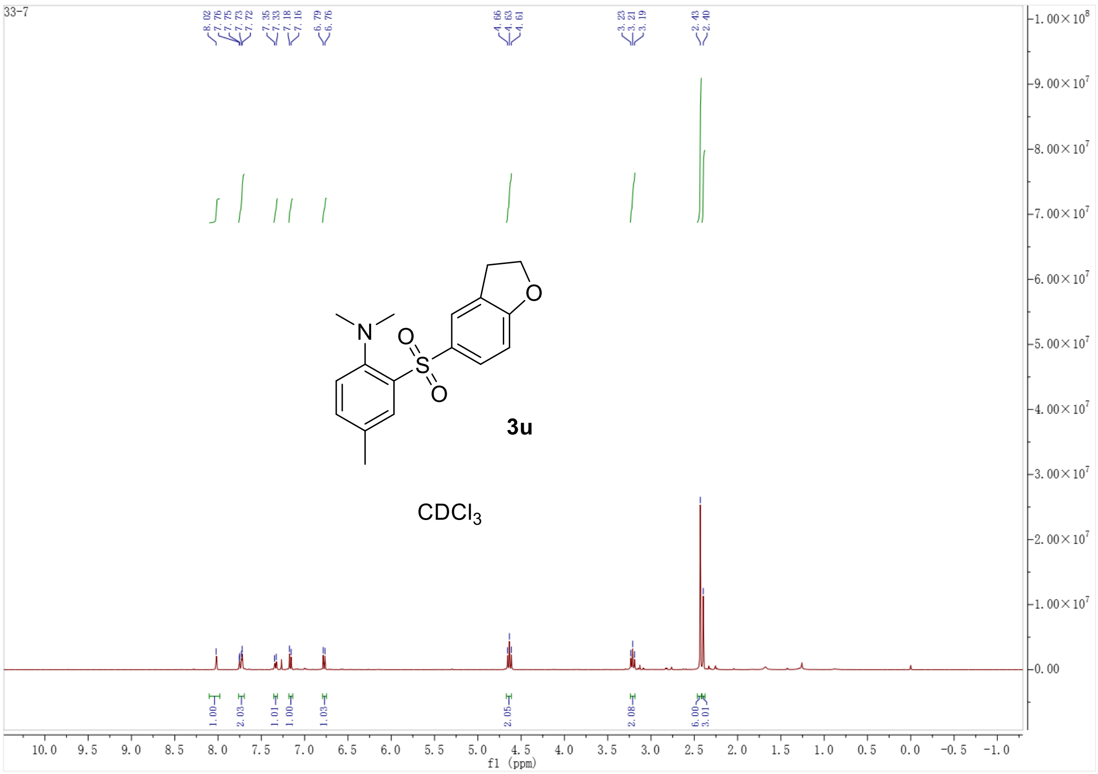
33-7
8.02
7.76
7.75
7.73
7.72
7.35
7.33
7.18
7.16
6.79
6.76
4.66
4.63
4.61
3.23
3.21
3.19
2.43
2.40
3u
CDCl3
1.00
2.03
1.01
1.00
1.03
2.05
2.08
6.00
3.01
f1 (ppm)

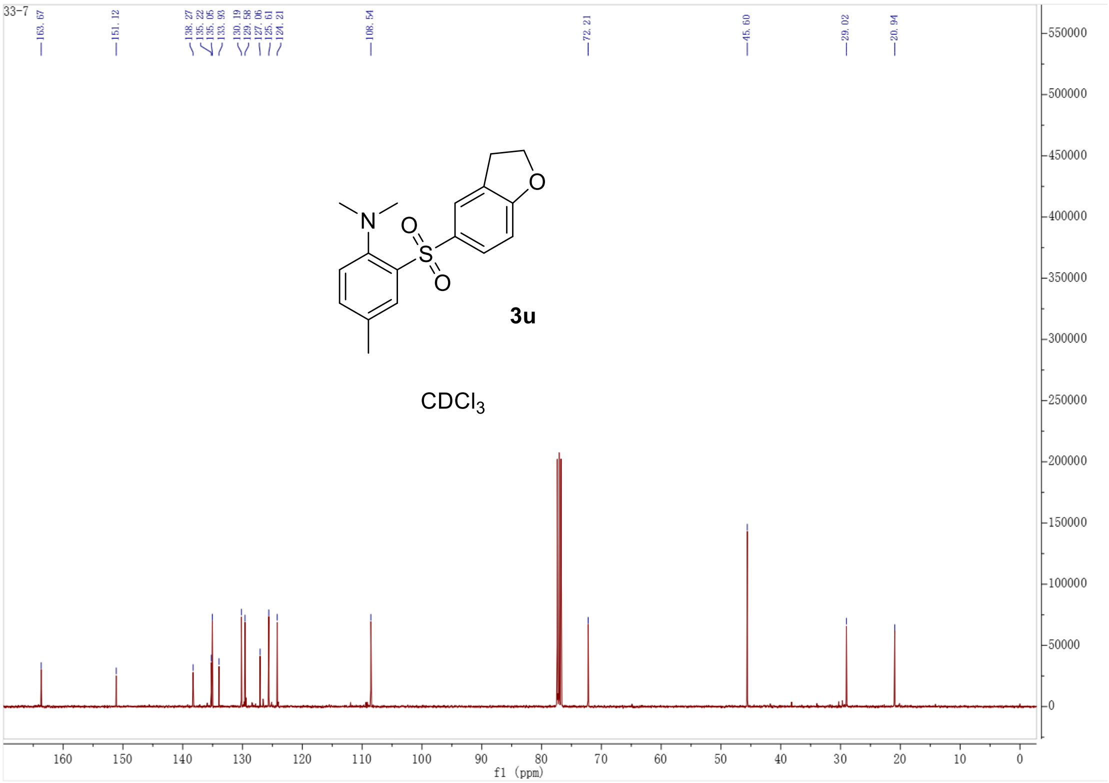
33-7
163.67
151.12
138.27
135.22
135.05
133.93
130.19
129.58
127.06
125.61
124.21
108.54
72.21
45.60
29.02
20.94
3u
CDCl3
f1 (ppm)

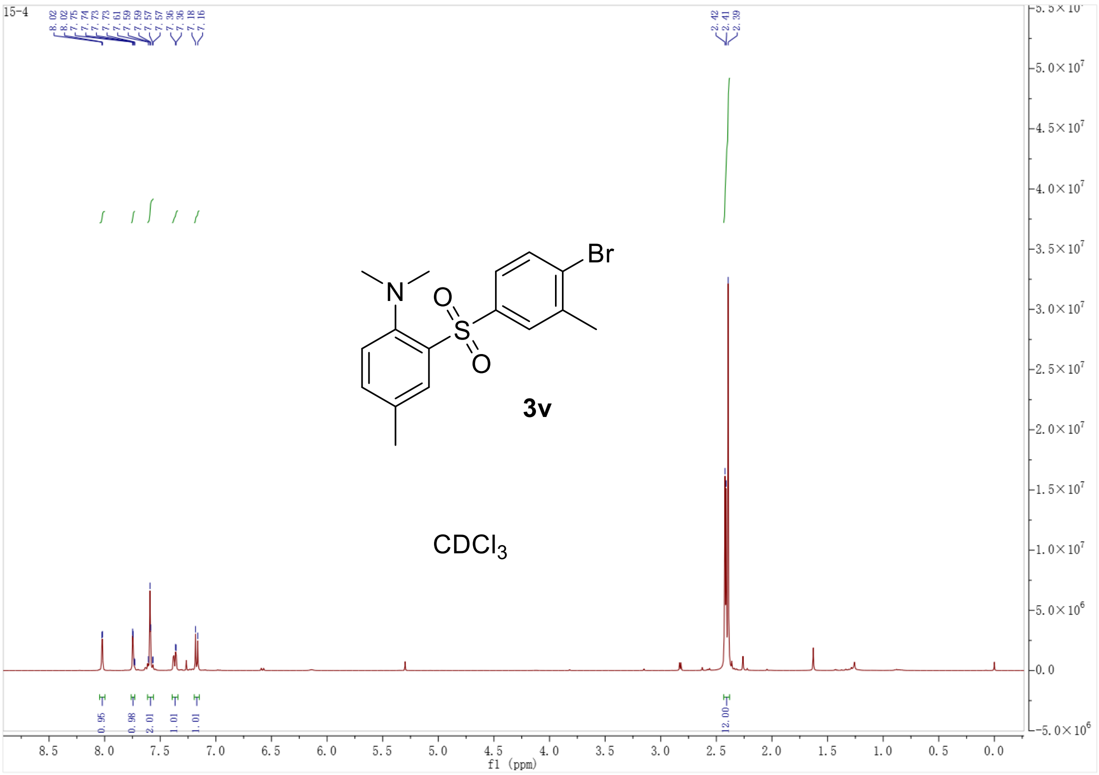
15-4
8.02
8.02
7.75
7.74
7.73
7.73
7.61
7.59
7.57
7.57
7.36
7.36
7.18
7.16
2.42
2.41
2.39
Br
N
O
S
O
3v
CDCl3
0.95
0.98
2.01
1.01
1.01
12.00
f1 (ppm)

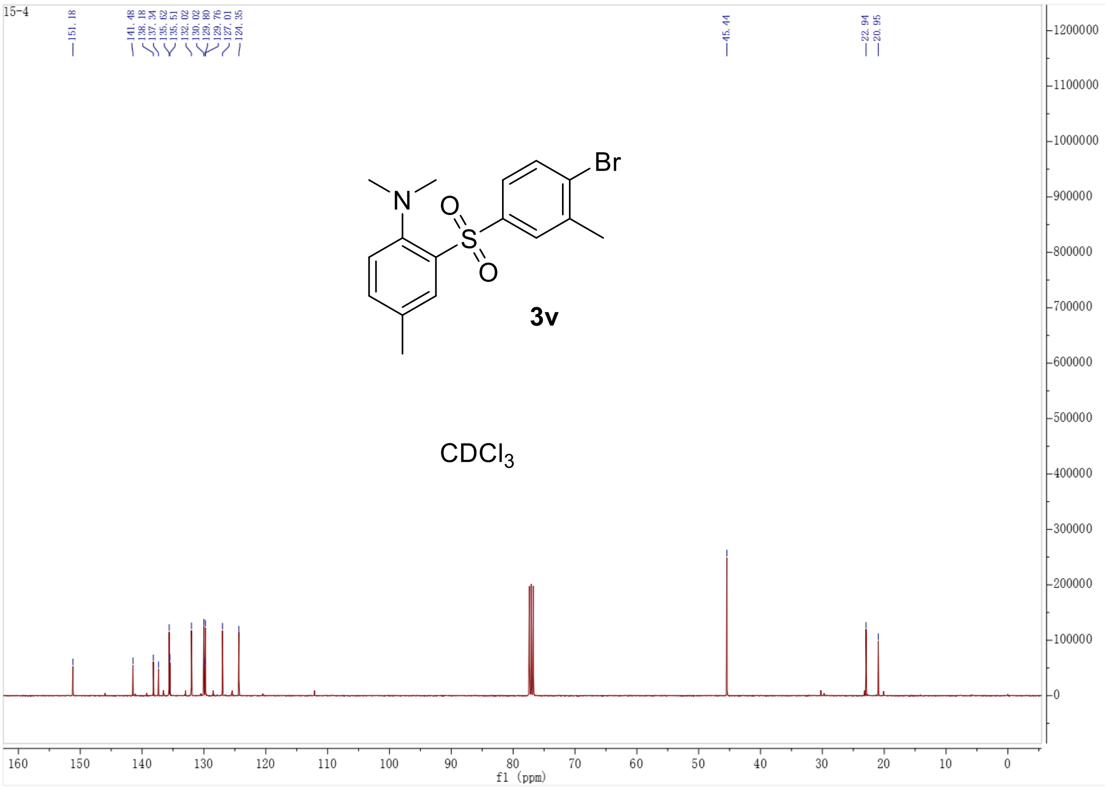
15-4
151.18
141.48
138.18
137.34
135.62
135.51
132.02
130.02
129.80
129.76
127.01
124.35
45.44
22.94
20.95
Br
N
O
S
O
3v
CDCl3
f1 (ppm)

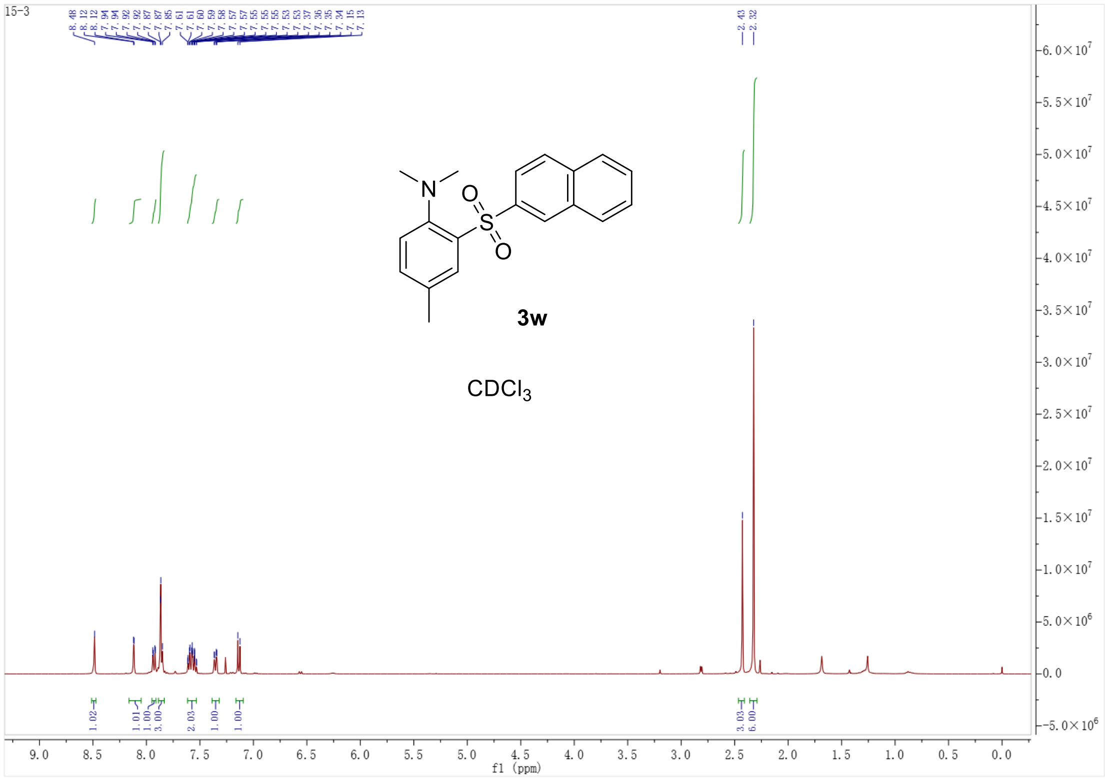
15-3
8.48 8.12 8.12 7.94 7.94 7.92 7.92 7.87 7.87 7.85 7.61 7.61 7.60 7.59 7.58 7.57 7.57 7.55 7.55 7.55 7.53 7.53 7.37 7.36 7.35 7.34 7.15 7.13
2.43 2.32
3w
CDCl3
1.02 1.01 1.00 3.00 2.03 1.00 1.00
3.03 6.00
f1 (ppm)

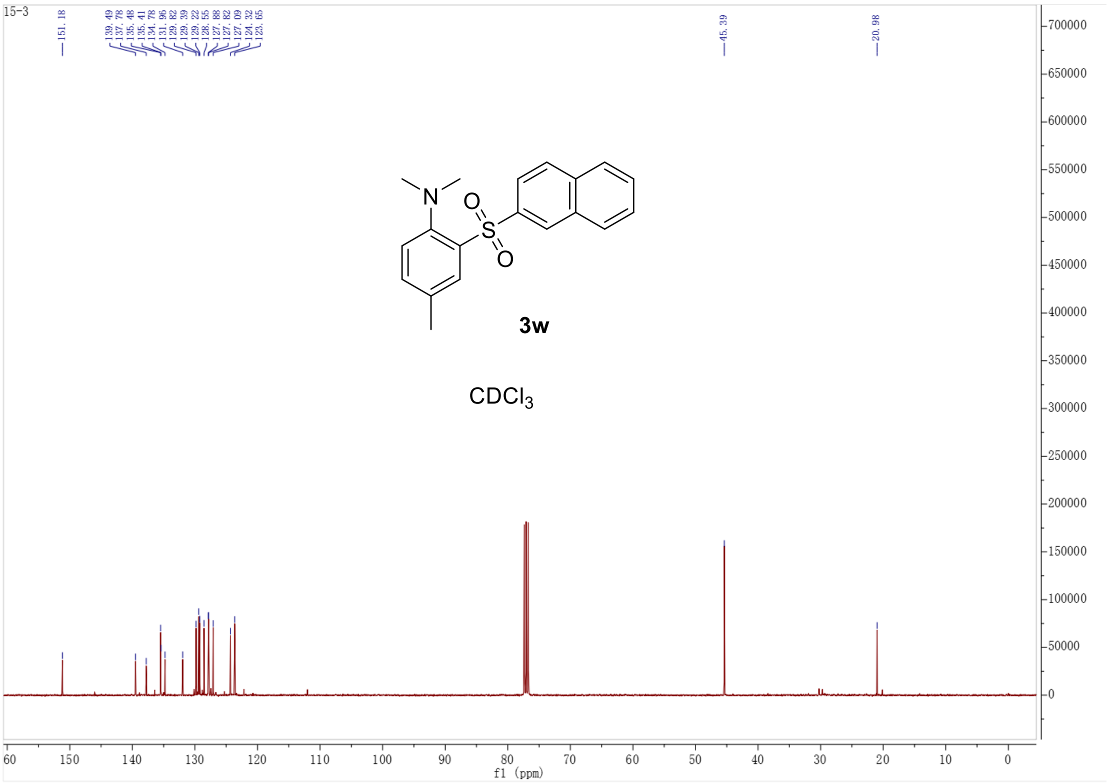
15-3
151.18
139.49 137.78 135.48 135.41 134.78 131.96 129.82 129.39 129.22 128.55 127.88 127.82 127.09 124.32 123.65
45.39
20.98
3w
CDCl3
f1 (ppm)

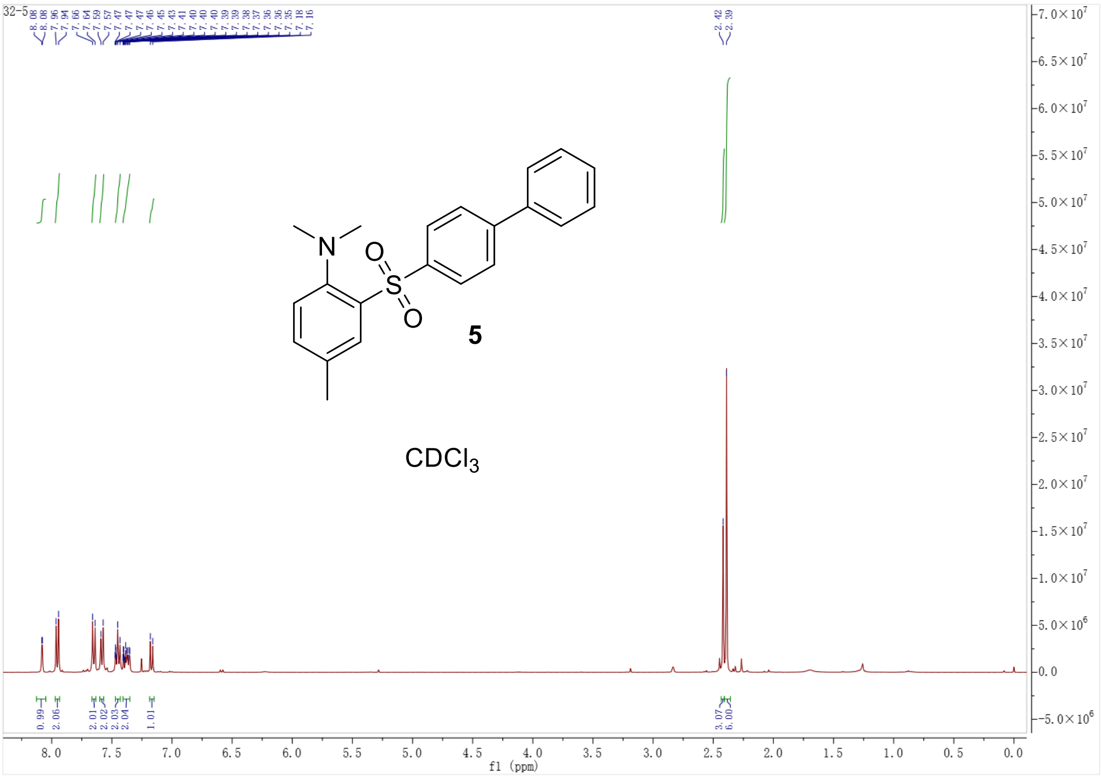
32-5
8.08 8.08 7.96 7.94 7.66 7.64 7.59 7.57 7.47 7.47 7.47 7.46 7.45 7.43 7.41 7.40 7.40 7.39 7.39 7.39 7.38 7.37 7.36 7.36 7.35 7.18 7.16
2.42 2.39
N
O
S
O
5
CDCl3
0.99 2.06 2.01 2.02 2.03 2.04 1.01
3.07 6.00
f1 (ppm)

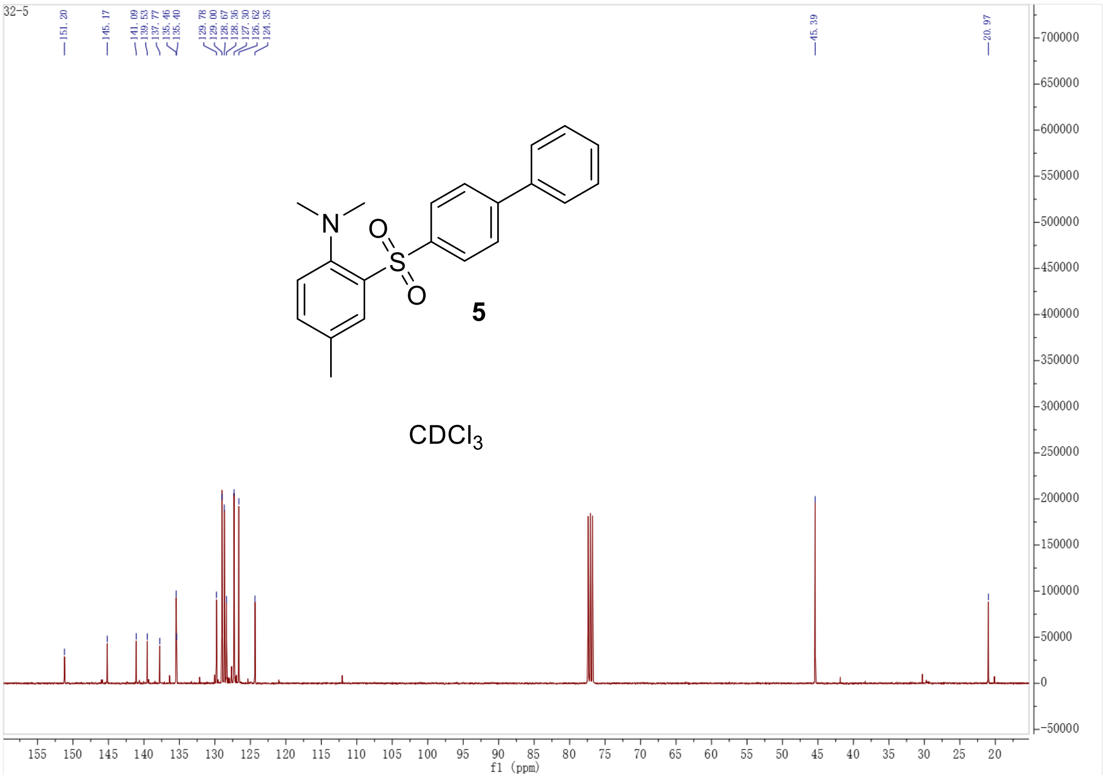
32-5
151.20
145.17
141.09 139.53 137.77 135.46 135.40
129.78 129.00 128.67 128.36 127.30 126.62 124.35
45.39
20.97
N
O
S
O
5
CDCl3
f1 (ppm)
